# Phylogeny, Pathogenicity, and Transmission of H5N1 Avian Influenza Viruses in Chickens

**DOI:** 10.3389/fcimb.2017.00328

**Published:** 2017-07-19

**Authors:** Jin Cui, Nannan Qu, Yang Guo, Lan Cao, Siyu Wu, Kun Mei, Hailiang Sun, Yiliang Lu, Zhifeng Qin, Peirong Jiao, Ming Liao

**Affiliations:** ^1^Animal Infectious Diseases Laboratory, College of Veterinary Medicine, South China Agricultural University Guangzhou, China; ^2^National and Regional Joint Engineering Laboratory for Medicament of Zoonosis Prevention and Control Guangzhou, China; ^3^Key Laboratory of Zoonosis, Ministry of Agriculture Guangzhou, China; ^4^Guangzhou Center for Disease Control and Prevention Guangzhou, China; ^5^Animal & Plant Inspection and Quarantine Technology Center of Shenzhen Entry-Exit Inspection and Quarantine Bureau of China Shenzhen, China; ^6^Department of Biotechnology, College of Life Sciences and Oceanography, Shenzhen University Shenzhen, China

**Keywords:** H5N1, highly pathogenic avian influenza virus, phylogeny, pathogenicity, transmission, chicken

## Abstract

We analyzed five H5N1 avian influenza viruses (AIVs) isolated from different birds in 2012 in China. Based on whole-genome sequences, we divided the viruses into four genotypes. The DKE26, GSE43, and DKE53 viruses belonged to Genotypes 1–3, respectively. The CKE93 and CKE96 viruses were classified into Genotype 4. Genotypes 1–3 correspond to the viruses containing the HA gene of clade 2.3.2, and Genotype 4 is the virus that bears the HA gene of clade 7.2. To better understand the pathogenicity and transmission of the viruses, we infected chickens with 10^3^ EID_50_/0.1 ml GSE43 (clade 2.3.2) or CKE93 (clade 7.2) virus. Our results revealed that 6 of 7 specific-pathogen-free (SPF) chickens inoculated with GSE43 virus were dead before 7-day post-infection, but all the SPF chickens inoculated with CKE93 virus survived the infection. Both the GSE43 and CKE93 viruses replicated systemically in chickens. The virus titers of GSE43 virus in tested organs were obviously higher than those of CKE93 virus. Our results revealed that the pathogenicity and replication of GSE43 in chickens was much higher than those of CKE93. The GSE43 virus could transmit between chickens, but the CKE93 could not transmit between chickens by naïve contact. Therefore, different clades of H5N1 AIVs possessed variable pathogenicities and transmission abilities among chickens. Our study contributes to knowledge of pathogenic variations of prevalent H5N1 viruses.

## Introduction

Avian influenza viruses (AIVs) are single-stranded negative-sense RNA viruses belonging to the Orthomyxoviridae family (Webster et al., 1992). In 1996, the first H5N1 AIVs were found in sick geese in Guangdong, and the H5N1 viruses were divided into highly pathogenic avian influenza viruses (HPAIVs) and low pathogenic avian influenza viruses (LPAIVs) (Li et al., 2006). The H5N1 HPAIVs infected 18 humans in Hong Kong in 1997, six of whom died (Subbarao et al., 1998). Till February 14, 2017, the World Health Organization (WHO) reported that there have been 856 humans infected with H5N1 HPAIVs in 16 countries; 452 of these patients have died (WHO, 2017). Since the beginning of 2004, there had been significant outbreaks of H5N1 AIVs infection involving multiple farm flocks in more than 20 provinces in China (Chen, 2009). It was clear that H5N1 HPAIVs could cause enormous economic losses and posed a serious public health threat.

H5N1 HPAIVs could be perpetuated in birds or mammals. Although the natural reservoir for AIVs was thought to be waterfowls, some viruses were highly pathogenic to domestic poultry (Chen et al., 2006). Moreover, H5N1 HPAIVs could be transmitted from waterfowls to mammalian and domestic poultry (Webster et al., 1992). In China, the H5N1 HPAIVs transmitted between aquatic birds and domestic poultry had contributed to the genetic diversity of the circulated viruses in domestic poultry (Duan et al., 2008; Vijaykrishna et al., 2008). H5N1 HPAIVs were classified into 10 clades (0–9) and several second clades by H5 HA nomenclature (World Health Organization/World Organization for Animal Health/Food and Agriculture Organization (WHO/OIE/FAO) H5N1 Evolution Working Group, 2014). The H5N1 viruses including clade 2.2, 2.3.2, 2.3.4, 4, 7, and 9 had been co-circulating from 2005 to 2006 in China, while the circulated viruses in domestic birds and waterfowls during 2007–2009 in China belonged to clades 2.3.2, 2.3.4, and 7 (Jiang et al., 2010). Several previous studies suggested that the pathogenicity of clade 2.3.2 viruses to waterfowls were increasing (Sakoda et al., 2010). Since 2005, H5N1 HPAIVs of clade 7 have occurred in chickens in northern China. The clade 7.2 viruses broke out in China in 2008 (e.g., in the Ningxia and Jiangsu regions), and caused a significant number of chicken deaths (WHO, 2012). To understand the transmission and pathogenicity of the H5N1 viruses in chicken, we chose five viruses belonging to various clades isolated from different birds.

## Materials and methods

### Viruses

The five H5N1 HPAIVs A/Duck/China/E26/2012(H5N1) (DKE26), A/Goose/China/E43/2012(H5N1) (GSE43), A/Duck/China/E53/2012(H5N1) (DKE53), A/Chicken/China/E93/2012(H5N1) (CKE93), and A/Chicken/China/E96/2012(H5N1) (CKE96) were obtained from swabs of different poultry in markets in 2012. Nine- to ten-days-old specific-pathogen-free (SPF) embryonated chicken eggs were used to purify and propagate the swabs by three rounds of limiting dilution method (Jiao et al., 2014; Yuan et al., 2014). The viruses were collected from allantoic fluids of multiple eggs. Values of 50% egg infective doses (EID_50_) was calculated using the Behrens-Reed-Muench cumulant method (Thakur and Fezio, 1981). Virus isolation and purification were done in biosafety level 3 (BSL-3) facilities.

### Sequence analysis

The genome of the viruses was sequenced. Trizol LS Reagent (Invitrogen Life Technologies, Carlsbad, CA, USA) was used to extract viral RNA from allantoic fluid and reverse transcription was performed with MLV (Invitrogen Life Technologies, Carlsbad, CA, USA). PCR amplification used pfx (Invitrogen Life Technologies, Carlsbad, CA, USA). QIAquick PCR purification kit (Qiagen, Valencia, CA, USA) was used to purify the PCR products and automatic ABI Prism 3730 genetic analyzer (Applied Biosystems, Foster City, CA, USA) was used to sequence. Lasergene 7.1 (DNASTAR, Madison, WI, USA) was used to compile and edit the DNA sequences. The phylogenetic tree was generated by the distance-based neighbor-joining method using MEGA 5 (Sinauer Associates, Inc., Sunderland, MA, USA). Bootstrap analysis with 1,000 replicates was used to assess the reliability of the trees. The genetic distance and horizontal distances were proportional. The nucleotide sequences in this study are available from NCBI GenBank (MF116309–MF116348).

### Pathogenicity and transmission

Four-week-old SPF White Leghorn chickens (*n* = 20) were allocated into two groups of 10 animals per group and housed in isolator cages. Seven chickens of the GSE43 or CKE93 group were inoculated intranasally with 10^3^ EID_50_/0.1 ml of GSE43 or CKE93 virus, respectively (Yuan et al., 2014). Contact group cohabited with those animals inoculated with the GSE43 or CKE93 virus were three chickens inoculated with 0.1 ml phosphate buffered saline (PBS). Significance of differences in survival was tested by Log-Rank analysis. Clinical symptoms of the chickens were observed for 14 days. Viral replication in the lungs, kidneys, brain, heart, spleen and liver of three infected chickens in each group were detected on day post-infection (DPI) 3. So were done on dead chickens. On 3, 5, 7, 9, and 11 DPI, cloacal and oropharyngeal swabs were collected from chickens and suspended in 1 ml PBS. All of the tissues and swabs were collected and titrated for virus infectivity in eggs. In accordance with the biosafety committee of South China Agriculture University protocols, the animal infected experiments were done in ABSL-3 facilities. The animal handling was done by the experimental animal administration and the ethics committee of South China Agriculture University guidelines.

## Results

### Phylogenetic analysis of the H5N1 viruses

**Figure 1:**
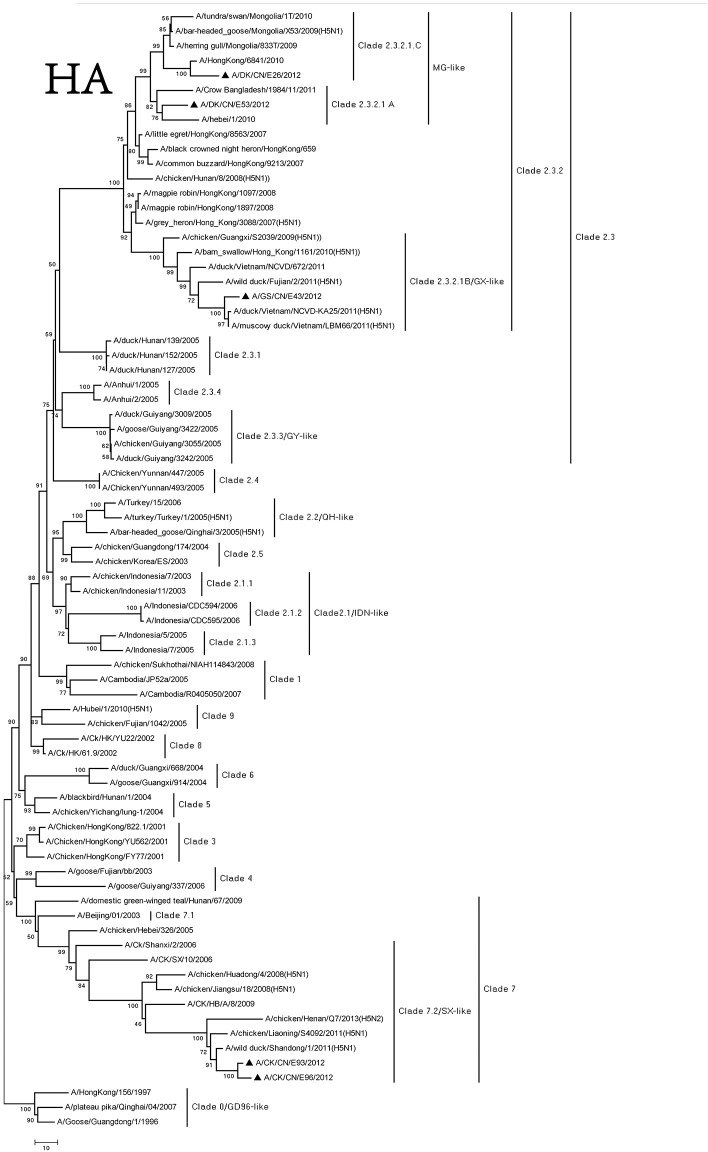
Phylogenetic analysis of HA. HA(A): nucleotides (nt) 29–1,732. Triangles indicate viruses characterized in this study, other virus sequence were downloaded from GenBank. QH, Qinghai; VN, Vietnam; IDN, Indonesia; GY, Guiyang; MG, Mongolia; GX, Guangxi; HD, Huadong; SX, Shanxi.

The genome of the viruses was sequenced to determine the molecular characteristic of them. We compared their sequences and those of the representative H5N1 viruses got from NCBI GenBank (Supplementary Table 5). Based on antigenic characteristics by the WHO, the HA genes of DKE26, GSE43, and DKE53 were classified to clade 2.3.2. The DKE53 could further be divided into clade 2.3.2.1.A. The DKE26 could further belong to 2.3.2.1.C. The GSE43 could further be classified into 2.3.2.1.B. The HA gene of other two viruses (CKE93 and CKE96) belonged to clade 7.2 (Figure 1). Compared with the GSGD1/96 virus nucleotide, the DKE26, GSE43, DKE53, CKE93, and CKE96 nucleotide similarities were 92.1, 91.3, 92.8, 91.8, and 91.6, respectively. We divided the HA genes of our isolates phylogenetically into three groups. The HA genes of the DKE26 and DKE53 viruses belonged to the MG-like group, and the HA gene of the GSE43 virus was classified into the GX-like group. The HA genes of the CKE93 and CKE96 viruses were clustered into the SX-like group. The HA genes similarity in groups were over 97%, and between the groups were <96%.

**Figure 2:**
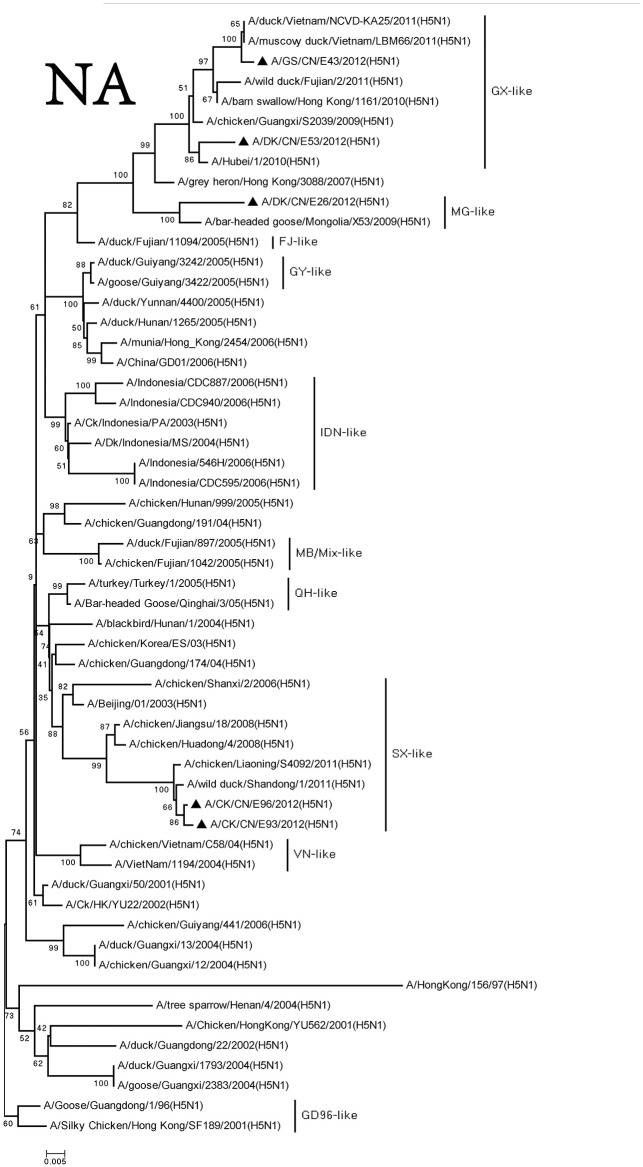
Phylogenetic analysis of NA. NA: nt19–1,380. Except our isolate, other virus sequences were downloaded from GenBank. QH, Qinghai; VN, Vietnam; IDN, Indonesia; GY, Guiyang; MG, Mongolia; GX, Guangxi; HD, Huadong; SX, Shanxi.

We retrieved complete NA sequences from NCBI GenBank to compare to the viruses containing the N1 gene. The NA genes of these viruses were divided into three groups, and the similarity among the three groups was <97%. The NA gene of the DKE26 virus belonged to the MG-like group. The NA genes of the GSE43 and DKE53 viruses were divided into the GX-like group. The NA genes of the CKE93 and CKE96 viruses were clustered to the SX-like group (Figure 2).

**Figure 3:**
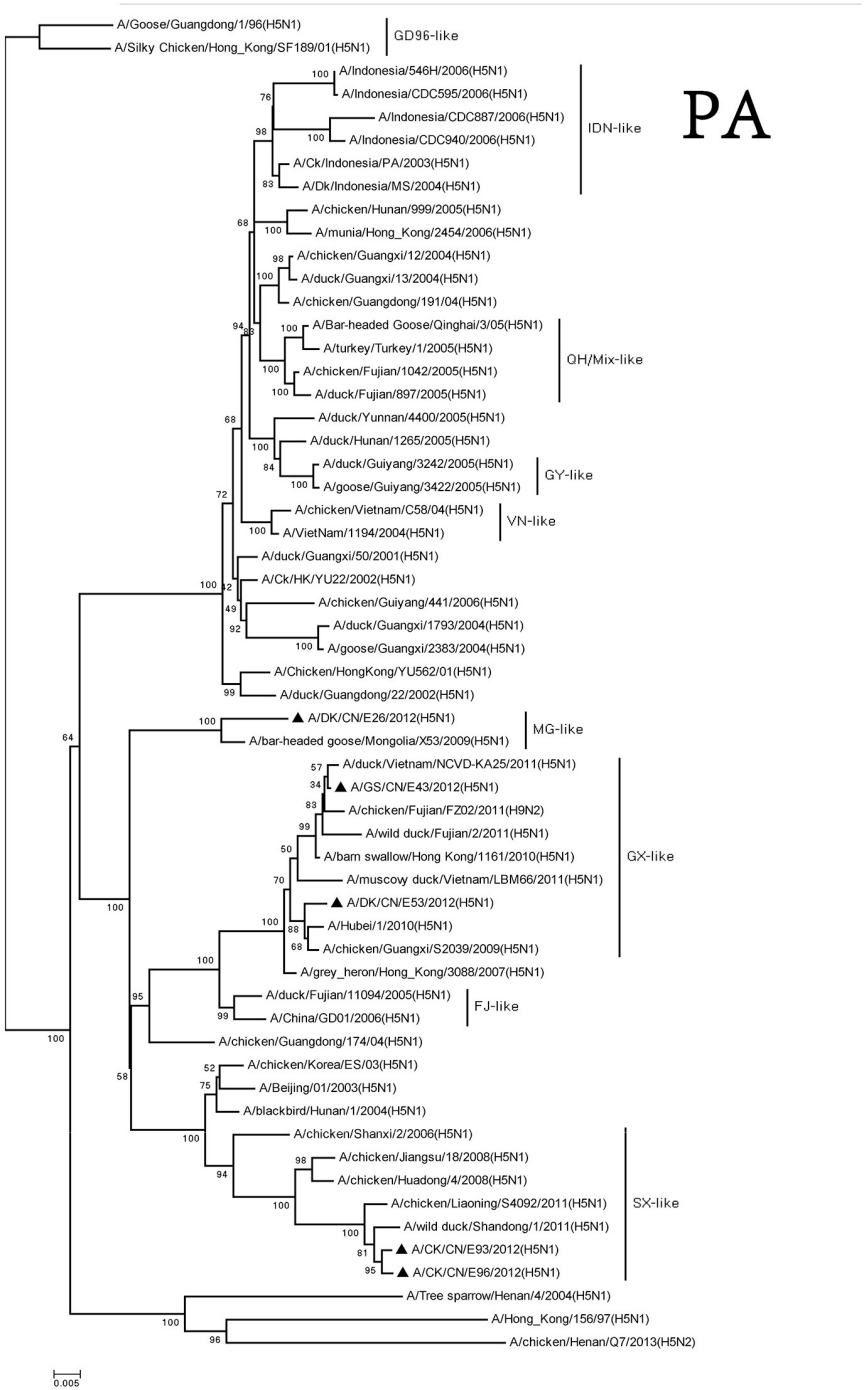
Phylogenetic analysis of PA. PA: nt 25–2,175. Except our isolate, other virus sequences were downloaded from GenBank. QH, Qinghai; VN, Vietnam; IDN, Indonesia; GY, Guiyang; MG, Mongolia; GX, Guangxi; HD, Huadong; SX, Shanxi.

The PA genes of these viruses were clustered into three groups. The MG-like group included of DKE26 virus, and the GX-like group contained GSE43 and DKE53 viruses. The CKE93 and CKE96 viruses were classified into the SX-like group. The PA genes of MG-like group viruses were <94% similarity with the GX-like group and the SX-like group viruses (Figure 3).

**Figure 4:**
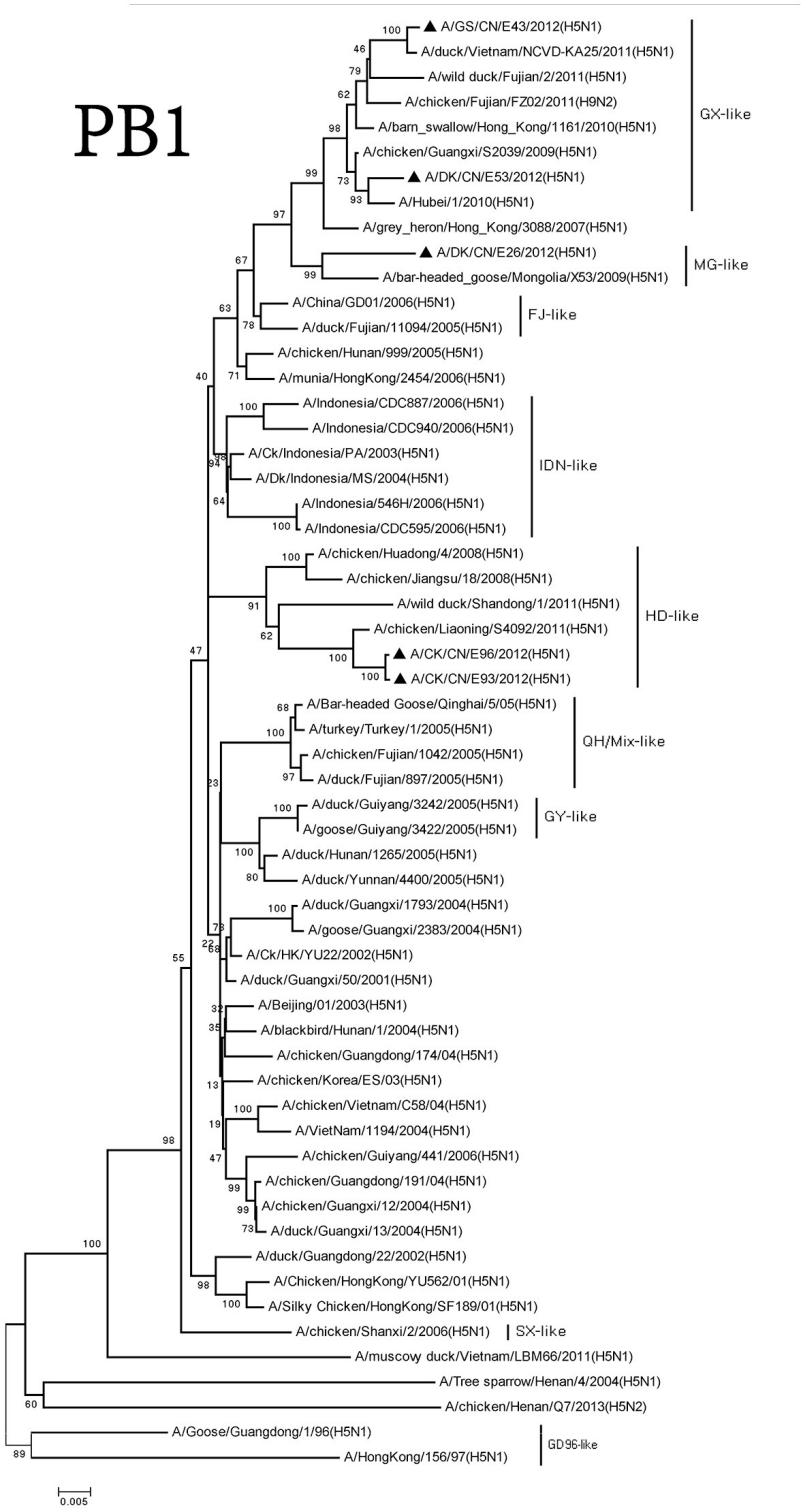
Phylogenetic analysis of PB1. PB1: nt 25–2,298. Except our isolate, other virus sequences were downloaded from GenBank. QH, Qinghai; VN, Vietnam; IDN, Indonesia; GY, Guiyang; MG, Mongolia; GX, Guangxi; HD, Huadong; SX, Shanxi.

We classified the PB1 genes of these H5N1 viruses into three groups. The PB1 gene of the DKE26 virus clustered to the MG-like group. The PB1 genes of the GSE43 and DKE53 viruses belonged to the GX-like group. The PB1 genes of the CKE93 and CKE96 viruses were classified into the SX-like group. The PB1 genes of SX-like group viruses were <95% similarity with other three groups, and the similarity among the SX-like group and the MG-like group ranged between 96 and 98% (Figure 4).

**Figure 5:**
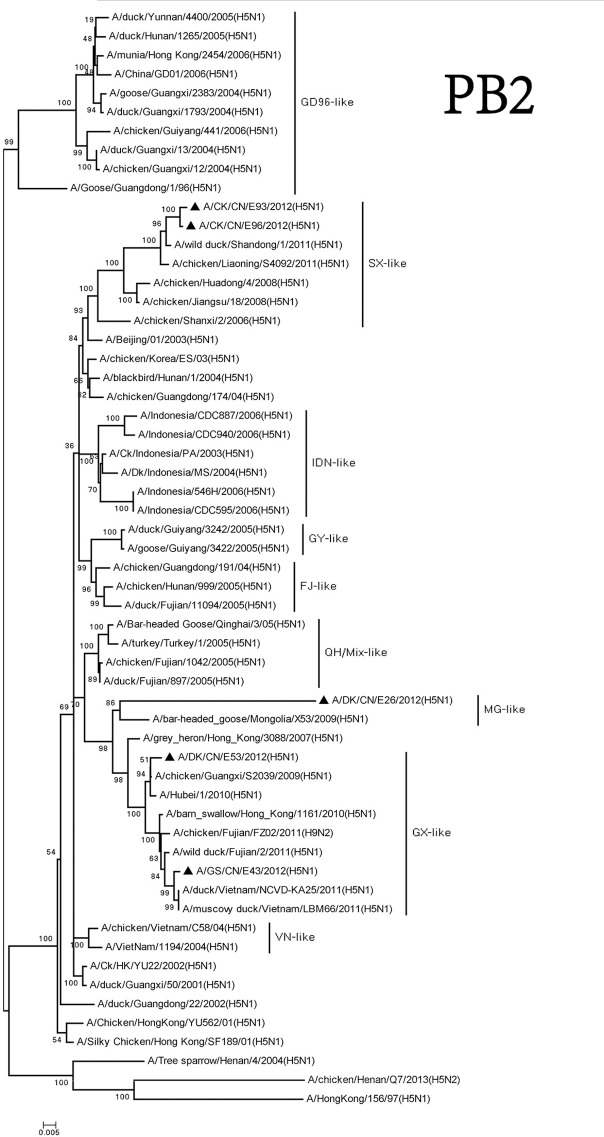
Phylogenetic analysis of PB2. PB2: nt 28–230. Except our isolate, other virus sequences were downloaded from GenBank. QH, Qinghai; VN, Vietnam; IDN, Indonesia; GY, Guiyang; MG, Mongolia; GX, Guangxi; HD, Huadong; SX, Shanxi.

The PB2 genes of these viruses consisted of three groups. The MG-like group included the PB2 gene of the DKE26 virus. The GX-like group contained the PB2 gene of the GSE43 and DKE53 viruses. The PB2 genes of the CKE93 and CKE96 viruses belonged to the SX-like group. The PB2 genes of the viruses among the three groups shared <93% similarity with one other (Figure 5).

**Figure 6:**
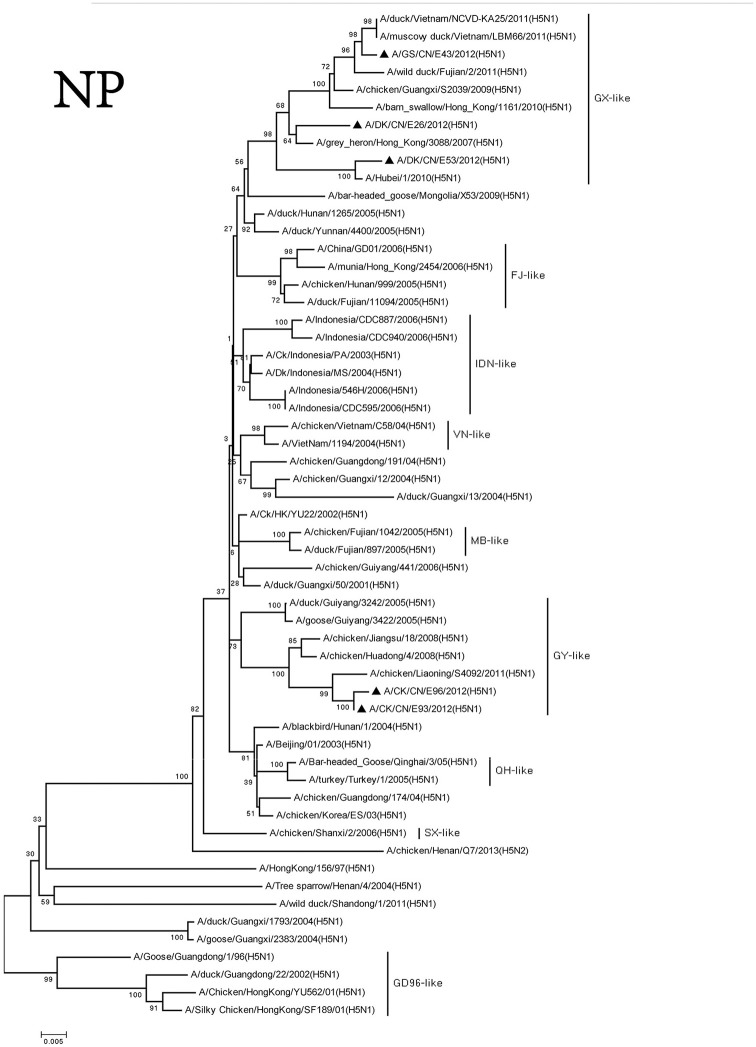
Phylogenetic analysis of NP. NP: nt46–1,542. Except our isolate, other virus sequences were downloaded from GenBank. QH, Qinghai; VN, Vietnam; IDN, Indonesia; GY, Guiyang; MG, Mongolia; GX, Guangxi; HD, Huadong; SX, Shanxi.

We divided the NP genes of these viruses into two groups. The NP genes of the DKE26, GSE43, and DKE53 viruses derived from GX-like viruses. The NP genes of the CKE93 and CKE96 viruses were clustered into GY-like viruses. The NP genes of the viruses in the GX-like group shared <95% similarity with those viruses in the GY-like group (Figure 6).

**Figure 7:**
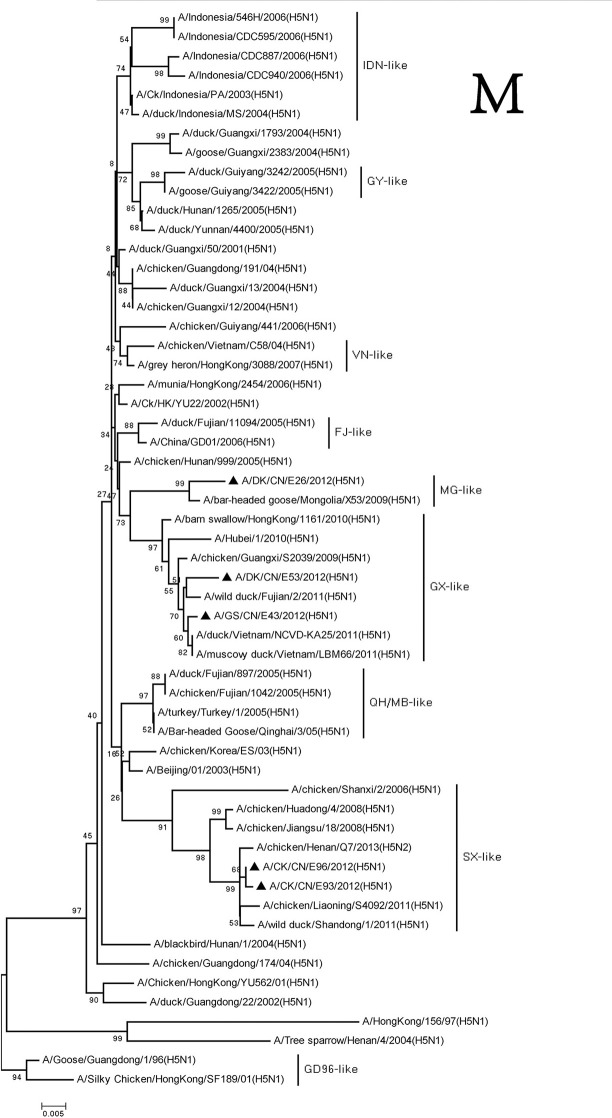
Phylogenetic analysis of M. M: nt26–795. Except our isolate, other virus sequences were downloaded from GenBank. QH, Qinghai; VN, Vietnam; IDN, Indonesia; GY, Guiyang; MG, Mongolia; GX, Guangxi; HD, Huadong; SX, Shanxi.

**Figure 8:**
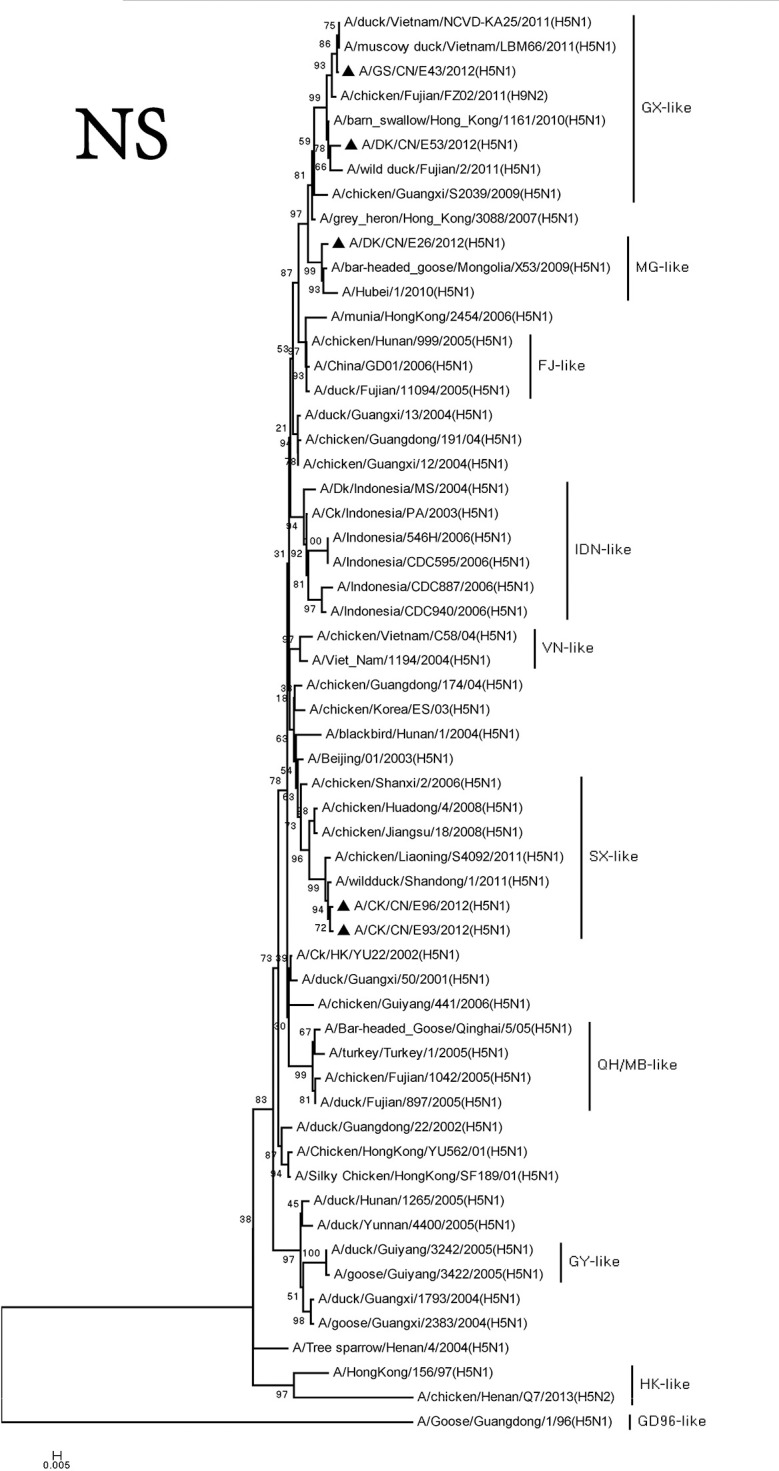
Phylogenetic analysis of NS. NS: nt27–678. Except our isolate, other virus sequences were downloaded from GenBank. QH, Qinghai; VN, Vietnam; IDN, Indonesia; GY, Guiyang; MG, Mongolia; GX, Guangxi; HD, Huadong; SX, Shanxi.

The M and NS genes of the viruses were divided into three groups. The M and NS genes of the DKE26 virus belonged to the MG-like group. The M and NS genes of the GSE43 and DKE53 viruses were clustered into the GX-like group. The M and NS genes of the CKE93 and CKE96 viruses were classified into the SX-like viruses, respectively (Figures 7, 8).

Based on genomic diversity, we here classified the viruses into four genotypes (Table 1). The H5N1 viruses of Genotypes 1–3 were isolated from Waterfowl, and Genotype 4 contained viruses from chickens. Genotypes 1–3 contained the HA gene of clade 2.3.2, and Genotype 4 contained the virus that bore the HA gene of clade 7.2, which was originally detected in chickens in China.

**Table 1:** Genotypic evolution of the H5N1 viruses isolated in China in 2012.

**Virus**	**Group of each gene segment in the phylogenetic tree**^**a**^								**Genotype**	**Species**
	**HA**	**NA**	**PB2**	**PB1**	**PA**	**NP**	**M**	**NS**		
A/DK/CN/E26/2012	MG	MG	MG	MG	MG	GX	MG	MG	1	DK
A/GS/CN/E43/2012	GX	GX	GX	GX	GX	GX	GX	GX	2	GS
A/DK/CN/E53/2012	MG	GX	GX	GX	GX	GX	GX	GX	3	DK
A/CK/CN/E93/2012	SX	SX	SX	HD	SX	GY	SX	SX	4	CK
A/CK/CN/E96/2012	SX	SX	SX	HD	SX	GY	SX	SX	4	CK

a *The eight gene segments are indicated at the top of each bar. The number in each bar shows the group of genes indicated in Figures 1–8*.

### Molecular characterization of the H5N1 viruses

A series of basic amino acids at the cleavage site of the HA (–RRRKR↓G–) were found in all the five viruses, which was characteristic of HPAIVs (Nobusawa et al., 1991; Belser et al., 2009). The amino acid residues Q226 and G228 in HA showed that the viruses preferentially bound to the AIVs receptor (Ha et al., 2001). We found three potential N-linked glycosylation sites in HA1 (27, 39, and 301 or 302) and two in HA2 (499 or 500 and 558 or 559) in the five viruses. An extra potential N-linked glycosylation site in HA1 (181) was found in DKE26, GSE43, and DKE53 viruses, and another two extra potential N-linked glycosylation site in HA1 (156 and 209) were found in the GSE43 virus. Five extra potential N-linked glycosylation sites in HA1 (88, 142, 155, 178, and 251) were found in CKE93 and CKE96 viruses (Table 2).

**Table 2:** Cleavage site and potential glycosylation sites in HA of the five H5N1 HPAIVs.

**Strains**	**Cleavage site**	**Potential glycosylation sites**													
	341–361/342–347	**27**	**39**	**88**	**142**	**155**	**156**	**178**	**181**	**209**	**251**	**288/289**	**301/302**	**499/500**	**558/559**
	−RRRKR/G–	**NST**	**NVT**	**NVS**	**NTS**	**NPS**	**NSS**	**NYT**	**NNT**	**NPT**	**NDT**	**NCS**	**NSS**	**NGT**	**NGS**
DKE26	+^a^	+	+	−^b^	−	−	−	−	+	−	−	−	+	+	+
GSE43	+	+	+	−	−	−	−	−	+	−	−	−	+	+	+
DKE53	+	+	+	−	−	−	+	−	+	+	−	−	+	+	+
CKE93	+	+	+	+	+	+	−	+	−	−	+	−	+	+	+
CKE96	+	+	+	+	+	+	−	+	−	−	+	−	+	+	+

a *The “+” means the amino acid sequences of glycosylation sites are same with list above*.

b *The “−” means the glycosylation sites are lost*.

The NA genes of five viruses had 20 amino acid deletions at positions 49–68. The mutations H274Y and N294S were not found in the viruses, which indicated the absence of antiviral drug-resistant residues (Gubareva et al., 2001).

Amantadine and rimantadine target the M2 protein, and single mutations in the trans-membrane domain of M2 (e.g., residues L26F, V27A/T, A30T/V, S31N/R, and G34E) could confer resistance to these drugs (Suzuki et al., 2003). An S31N mutation was observed in the M2 gene of CKE93 and CKE96 viruses, suggesting that the CKE93 and CKE96 viruses were not sensitive to this class of antiviral drugs. No amino acid substitutions were found in other residues.

The S314N mutation in the NP of H5N1 virus could cause a defect in nuclear localization at high temperature (Siboonnan et al., 2013). The mutation N319K in NP might influence pathogenicity (Gabriel et al., 2005), but no amino acid substitutions were found in these residues in the viruses.

The NS1 genes of the DKE26, GSE43, DKE53, CKE93, and CKE96 viruses had a five-amino-acid deletion at positions 80–84, which may contribute to increased virulence (Long et al., 2008). Previous studies had shown that P42S mutations may contribute to the virulence of H5N1 viruses in mice (Jiao et al., 2008); these mutations were observed the NS1 genes of the five viruses. An importation virulence factor of influenza A viruses was the PDZ-binding motif of NS1 (Liu et al., 2010). The sequences of the DKE26, DKE53, CKE93, and CKE96 viruses in the PDZ domain were ESEV, which also was found in the NS1 of 1918 pandemic virus and 1997–2003 H5N1 viruses (Jackson et al., 2008).

Previous study showed that the mutation T515A in PA gene contributes to the viruses transmission in ducks (Hulse-Post et al., 2007). The amino acid 224P in PA increased the replication of the virus in duck embryo fibroblasts (Song et al., 2011). The absence of the S224P and T515A mutations in the PA protein suggests poor replication of those viruses in ducks. The mutation R185K and C241Y in PA gene enhanced the growth capability of the viruses in human cells (Fan et al., 2014; Yamaji et al., 2015). The presence of 185R and 241C in the PA protein showed low growth capability of the viruses in human cell.

The presence of L13P and S678N mutations in the PB1 protein increases mammalian pathogenicity (Zell et al., 2007). Although the L13P mutation was present in the five isolates, the S678N mutation was not found, which indicates that those viruses did not acquire increased mammalian pathogenicity. A 90-residue PB1-F2 was observed in DKE26, DKE53, CKE93, and CKE96 viruses, but the GSE43 virus encoded a 57aa PB1-F2 protein.

The E627K substitution was absent in the PB2 protein of the GSE43, DKE53, CKE93, and CKE96 viruses, but it was present in the DKE26 virus. The presence of 701D and 714S in the PB2 protein of the five viruses suggested that the virus might not be capable of increased virulence in mammalian species.

### Pathogenicity of H5N1 HPAIVs in chickens

**Figure 9:**
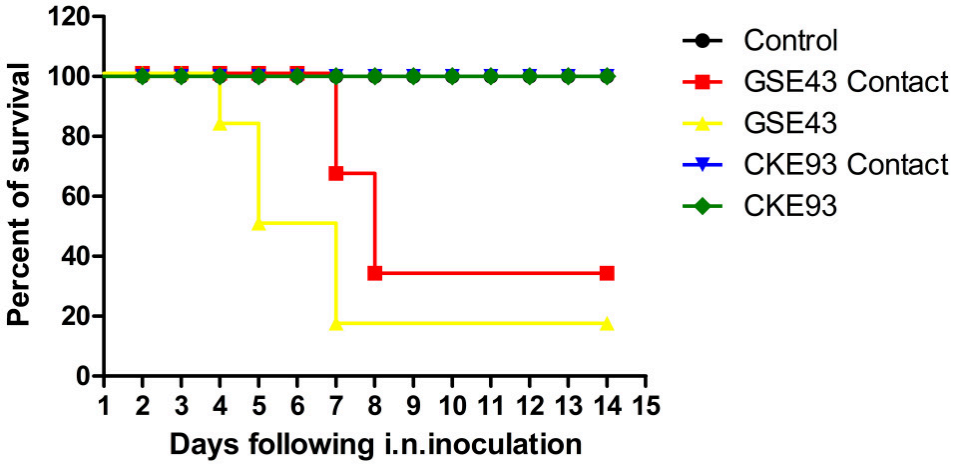
Lethality of the A/GS/CN/E43/2012 and A/CK/CN/E93/2012 viruses in SPF chicken. Death patterns of the SPF chicken infected with A/GS/CN/E43/2012 and A/CK/CN/E93/2012 viruses with the doses of 10^3^ EID_50_. Curves are significantly different (*P* < 0.05) by log-rank analysis.

The GSE43 of clade 2.3.2.1B and CKE93 of clade 7.2 viruses were obtained from swabs of gooses and chickens with apparent clinical symptoms. We focused on the two viruses to assess the pathogenicity of the H5N1 HPAIVs in chickens. SPF chickens were infected i.n. at 10^3^ EID_50_. The Mean Death Time (MDT) of SPF chickens inoculated with GSE43 was 4.6 days (83.3% mortality) (Figure 9). No chicken inoculated with CKE93 virus died during the observation period (Figure 9). Torticollis and neurological symptoms were observed among SPF chickens infected with GSE43. Only one SPF chicken with slight torticollis was observed in the CKE93-inoculated group. Therefore, the two viruses had various pathogenicities in chickens.

The GSE43 and CKE93 viruses replicated systemically in chickens, which could be found from all of the tested organs—including the heart, liver, spleen, lungs, kidneys, and brain—3 DPI. The virus titers of GSE43 virus in tested organs were obviously higher than those of CKE93 virus. The mean virus titers of the GSE43 in the heart, liver, spleen, lungs, kidneys and brain were 6.63, 8.63, 8.63, 7.75, 8.63, and 8.63 log_10_EID_50_, respectively. The virus titer of CKE93 virus in the heart, liver, spleen, lungs, kidneys and brain were 3.67, 3.00, 3.67, 4.00, 3.75, and 3.67 log_10_EID_50_, respectively (Table 3). All in all, the replication of GSE43 in chickens was much higher than that of CKE93.

**Table 3:** Replication and lethality in chickens of the H5N1 viruses after inoculated intranasally^a^.

**Strains**	**Clade**	**Titer (log_10_EID_50_)**		**Virus replication on 3 DPI (log_10_EID_50_/0.1ml)^b^ in**					
				**Heart**	**Liver**	**Spleen**	**Lung**	**Kidney**	**Brain**
GSE43	Clade 2.3.2.1.B	6.8	Infected^c^	6.63	8.63	8.63	7.75	8.63	8.63
			Contact^d^	6.63	8.63	8.63	8.00 ± 1.41	7.81 ± 1.15	7.81 ± 1.15
CKE93	Clade 7.2	7.3	Infected^c^	3.67	3.00	3.67	4.00	3.75	3.67
			Contact^d^	–	–	–	–	–	–

a *Four-week-old SPF chickens were inoculated intranasally (i.n.) with 10^3^ EID_50_ of E43 virus, 10^3^ EID_50_ of E93 virus in a volume of 100 μl, respectively; three chickens in each group were euthanized on 3 DPI, and virus titer was determined in samples of heart, liver, spleen, lung, kidney, and brain in eggs*.

b *For statistical analysis, a value of 1.5 was assigned if the virus was not detected from the undiluted sample in three embryonated hen eggs. Virus titers are expressed as means standard deviation in log10EID_50_/0.1 ml of tissue*.

c *Ducks inoculated with virus*.

d *Naive contact ducks housed with those inoculated*.

Oropharyngeal and cloacal swabs collected on 3, 5, 7, 9, and 11 DPI were used to detect viruses shedding from the inoculated chickens. The GSE43 virus could not be tested from oropharyngeal swabs, but it was detected from cloacal swabs 3 DPI. The GSE43 virus was not detected after 3 DPI. The virus shedding of CKE93 were not detected from swabs of inoculated SPF chickens (Table 4). In conclusion, the virus shedding time of chickens inoculated with GSE43 was longer than that of chickens infected with CKE93.

**Table 4:** Virus titers in cloacal and oropharyngeal swabs from SPF chicken.

**Strains**		**Days post-inoculation (log_10_EID_50_/0.1 ml) ± SD^a^**									
		**3 Day**		**5 Day**		**7 Day**		**9 Day**		**11 Day**	
		**Oropharyngeal swabs**	**Cloacal swabs**	**Oropharyngeal swabs**	**Cloacal swabs**	**Oropharyngeal swabs**	**Cloacal swabs**	**Oropharyngeal swabs**	**Cloacal swabs**	**Oropharyngeal swabs**	**Cloacal swabs**
GSE43	Infected^b^	0/3	1/3	0/1	0/1	0/1	0/1	0/1	0/1	–	–
	Contact^c^	1/3	0/3	1/2	1/2	0/1	0/1	0/1	0/1	0/1	0/1
CKE93	Infected^b^	0/6	0/6	0/3	0/3	0/3	0/3	0/3	0/3	0/3	0/3
	Contact^c^	0/3	0/3	0/3	0/3	0/3	0/3	0/3	0/3	0/3	0/3

a *For statistical purposes, a value of 1.5 was assigned if virus was not detected from the undiluted sample in three embryonated hen's eggs (Sun et al., 2011)*.

b *Chickens inoculated with virus*.

c *Naive contact chickens housed with those inoculated*.

### Transmission of H5N1 HPAIVs in chickens

To understand the horizontal transmission of these two viruses, three SPF chickens were inoculated intranasally with 0.1 ml PBS as a naïve-contact group. These animals were then housed with chickens inoculated with the GSE43 and CKE93 viruses. We noted that two SPF chickens in the naïve-contact group, housed with inoculated GSE43 chickens, died 3 and 5 DPI. The last naïve-contact chicken seroconverted 14 DPI. The GSE43 virus could be detected from oropharyngeal swabs of naïve-contact chickens 3 and 5 DPI and from cloacal swabs 5 DPI. The GSE43 virus also replicated systemically in naïve-contact chickens; the mean virus titers in the heart, liver, spleen, lungs, kidneys and brain were 6.63, 8.63, 8.63, 8.00 ± 1.41, 7.81 ± 1.15, and 7.81 ± 1.15 log_10_EID_50_, respectively. All of the three naïve-contact chickens housed with CKE93 survived and virus shedding was not detected from swabs. The CKE93 virus was not detected in any tested organs in naïve-contact SPF chickens (Table 3).

Therefore, we found that the GSE43 virus could transmit between chickens by naïve contact. However, the CKE93 virus could not be transmitted to SPF chickens via naïve contact.

## Discussion

Since late 2003, thousands of wild birds and millions of poultry death have been caused by H5N1 HPAI viruses, especially in southern China and Southeast Asia. Despite substantial efforts to control infection rates in poultry, H5N1 HPAI has broken out in some Chinese provinces. Because the wild birds migrate and the domestic poultry move, the viruses might spread at any time. In 2004, the first clade 2.3.2 virus was isolated from a dead Chinese pond heron in Hong Kong; it is now widely distributed in Asia and in Eastern Europe. Therefore, viruses of clade 2.3.2 have spread geographically and evolved genetically (WHO, 2012). Viruses of clade 2.3.2.1 have been circulating widely in China since 2010 and may have been responsible for a new wave of cross-continental spreading from Asia to Europe (Smith et al., 2009; Jiang et al., 2010; Li et al., 2010). This clade was already widely distributed in chickens, ducks, geese, and wild birds (WHO, 2015). Since 2003, the clade 7 H5N1 HPAIVs has been spreading widely in Northern China. The viruses of clade 7.2 have been spreading in China and Vietnam since 2008 (WHO, 2011). From 2010 to 2013, the clade 2.3.2.1 and 7.2 H5N1 viruses were dominant in Southern China and other clades had occasionally been found (WHO, 2013). The circulated viruses had been responsible for significantly damaging the poultry industry. Therefore, it is necessary to understand the phylogeny, pathogenicity and transmission of the viruses from clade 2.3.2 and 7.2.

The previous reported that the clade 2.3.2 AIVs had reasserted with H9 viruses and the reassortment between clades 2.3.2.1c, 2.3.2.1b, and 2.3.2.1a virus had been found (Marinova-Petkova et al., 2016; Nguyen et al., 2016). Our results showed The HA gene of the Genotypes 1 virus was same as that of Genotype 3, but the other seven genes were different from Genotypes 2 and 3. The NA, PA, PB1, PB2, NP, M, and NS genes of the Genotype 2 and 3 viruses were quite similar, but their HA genes exhibited significant diversity (Table 1). So the Genotype 3 virus may be a reassortant from Genotype 1 and 2 viruses. The SX-like viruses were first detected in chickens from the Shanxi province in Northern China, but the origin of these viruses remains unclear. However, genomic analyses confirmed that the SX-like viruses reassorting with new HA, NA, and PB1 genes were newly introduced into poultry in China (Li et al., 2010). Now the clade 7.2 viruses had derived gene segments from viruses of clade 2.3.4 or H9N2 (Liu et al., 2016). Our results showed that the HA, NA, M, NS, PA, and PB1 genes of Genotypes 4 viruses belonged to SX-like group viruses, but the NP and PB1 genes belonged to GY-like group viruses (clade 2.3.3) and HD-like group viruses, respectively. So the clade 7.2 viruses may had been reassorted with clade 2.3.3 viruses. This finding showed that multiple clades viruses cocirculating in waterfowl may promote the reassortment of the viruses.

Previous studies have shown that both clade 2.3.2 and 7.2 viruses were highly pathogenic to chickens, and could transmit to the naive contact chickens (Yuan et al., 2014; Jiao et al., 2016). In our study, the GSE43 (clade 2.3.2) and CKE93 (clade 7.2) viruses from the different birds in 2012 were evaluated the pathogenicity of H5N1 HPAIVs in chickens. They could replicate systemically in chickens, but the virus titer of GSE43 virus was higher than that of the CKE93 virus. The pathogenicity of the CKE93 virus was much lower than that of the GSE43 virus. Especially, only the GSE43 virus could be transmitted between SPF chickens by naïve contact. Therefore, the H5N1 HPAIVs of clade 2.3.2 obtained from waterfowls were highly pathogenic to chickens and replicated systemically in chickens. And we found that the clade 7.2 virus could not be transmitted in chickens when the animals were inoculated with a low dose.

In conclusion, our findings revealed that clade 2.3.2 and 7.2 H5N1 viruses had varying levels of pathogenicity and transmission in chickens. Therefore, we should intensify virological surveillance of the H5N1 viruses to understand the antigenic and pathogenic variations of prevalent H5N1 viruses.

## Author contributions

JC and PJ designed this study, performed the experiment sand participated in the data collection and analysis. NQ, YG, LC, SW, KM, and YL assisted with animal experiment. JC and PJ drafted the manuscript. ML, HS, and ZQ participated in writing the discussion.

### Conflict of interest statement

The authors declare that the research was conducted in the absence of any commercial or financial relationships that could be construed as a potential conflict of interest.

## References

[B1] Belser J. A. Szretter K. J. Katz J. M. Tumpey T. M. (2009). Use of animal models to understand the pandemic potential of highly pathogenic avian influenza viruses. Adv. Virus Res. 73, 55–97. 10.1016/S0065-3527(09)73002-7 19695381

[B2] Chen H. (2009). Avian influenza vaccination: the experience in China. Int. Epizoot. 28, 267–274. 10.20506/rst.28.1.1860 19618631

[B3] Chen H. Li Y. Li Z. Shi J. Shinya K. Deng G.. (2006). Properties and dissemination of H5N1 viruses isolated during an influenza outbreak in migratory waterfowl in western China. J. Virol. 80, 5976–5983. 10.1128/JVI.00110-06 16731936 PMC1472608

[B4] Duan L. Bahl J. Smith G. J. Wang J. Vijaykrishna D. Zhang L. J.. (2008). The development and genetic diversity of H5N1 influenza virus in China, 1996-2006. Virology 380, 243–254. 10.1016/j.virol.2008.07.038 18774155 PMC2651962

[B5] Fan S. Hatta M. Kim J. H. Le M. Q. Neumann G. Kawaoka Y. (2014). Amino acid changes in the influenza A virus PA protein that attenuate avian H5N1 viruses in mammals. J. Virol. 88, 13737–13746. 10.1128/JVI.01081-14 25231317 PMC4248959

[B6] Gabriel G. Dauber B. Wolff T. Planz O. Klenk H. D. Stech J. (2005). The viral polymerase mediates adaptation of an avian influenza virus to a mammalian host. Proc. Natl. Acad. Sci. U.S.A. 102, 18590–18595. 10.1073/pnas.0507415102 16339318 PMC1317936

[B7] Gubareva L. V. Kaiser L. Matrosovich M. N. Soo-Hoo Y. Hayden F. G. (2001). Selection of influenza virus mutants in experimentally infected volunteers treated with oseltamivir. J. Infect. Dis. 183, 523–531. 10.1086/318537 11170976

[B8] Ha Y. Stevens D. J. Skehel J. J. Wiley D. C. (2001). X-ray structures of H5 avian and H9 swine influenza virus hemagglutinins bound to avian and human receptor analogs. Proc. Natl. Acad. Sci. U.S.A. 98, 11181–11186. 10.1073/pnas.201401198 11562490 PMC58807

[B9] Hulse-Post D. J. Franks J. Boyd K. Salomon R. Hoffmann E. Yen H. L.. (2007). Molecular changes in the polymerase genes (PA and PB1) associated with high pathogenicity of H5N1 influenza virus in mallard ducks. J. Virol. 81, 8515–8524. 10.1128/JVI.00435-07 17553873 PMC1951362

[B10] Jackson D. Hossain M. J. Hickman D. Perez D. R. Lamb R. A. (2008). A new influenza virus virulence determinant: the NS1 protein four C-terminal residues modulate pathogenicity. Proc. Natl. Acad. Sci. U.S.A. 105, 4381–4386. 10.1073/pnas.0800482105 18334632 PMC2393797

[B11] Jiang W. M. Liu S. Chen J. Hou G. Y. Li J. P. Cao Y. F.. (2010). Molecular epidemiological surveys of H5 subtype highly pathogenic avian influenza viruses in poultry in China during 2007-2009. J. Gen. Virol. 91, 2491–2496. 10.1099/vir.0.023168-0 20610668

[B12] Jiao P. Song H. Liu X. Song Y. Cui J. Wu S.. (2016). Pathogenicity, transmission and antigenic variation of H5N1 highly pathogenic avian influenza viruses. Front. Microbiol. 7:635. 10.3389/fmicb.2016.00635 27199961 PMC4858587

[B13] Jiao P. Tian G. Li Y. Deng G. Jiang Y. Liu C.. (2008). A single-amino-acid substitution in the NS1 protein changes the pathogenicity of H5N1 avian influenza viruses in mice. J. Virol. 82, 1146–1154. 10.1128/JVI.01698-07 18032512 PMC2224464

[B14] Jiao P. Wei L. Song Y. Cui J. Song H. Cao L. (2014). D701N mutation in the PB2 protein contributes to the pathogenicity of H5N1 avian influenza viruses but not transmissibility in guinea pigs. Front. Microbiol. 5:642 10.3389/fmicb.2014.00642 25505461 PMC4243574

[B15] Li Y. Shi J. Zhong G. Deng G. Tian G. Ge J.. (2010). Continued evolution of H5N1 influenza viruses in wild birds, domestic poultry, and humans in China from 2004 to 2009. J. Virol. 84, 8389–8397. 10.1128/JVI.00413-10 20538856 PMC2919039

[B16] Li Z. Jiang Y. Jiao P. Wang A. Zhao F. Tian G.. (2006). The NS1 gene contributes to the virulence of H5N1 avian influenza viruses. J. Virol. 80, 11115–11123. 10.1128/JVI.00993-06 16971424 PMC1642184

[B17] Liu H. Golebiewski L. Dow E. C. Krug R. M. Javier R. T. Rice A. P. (2010). The ESEV PDZ-binding motif of the avian influenza A virus NS1 protein protects infected cells from apoptosis by directly targeting Scribble. J. Virol. 84, 11164–11174. 10.1128/JVI.01278-10 20702615 PMC2953166

[B18] Liu L. Zeng X. Chen P. Deng G. Li Y. Shi J.. (2016). Characterization of Clade 7.2 H5 avian influenza viruses that continue to circulate in chickens in China. J. Virol. 90, 9797–9805. 10.1128/JVI.00855-16 27558424 PMC5068530

[B19] Long J. X. Peng D. X. Liu Y. L. Wu Y. T. Liu X. F. (2008). Virulence of H5N1 avian influenza virus enhanced by a 15-nucleotide deletion in the viral nonstructural gene. Virus Genes 36, 471–478. 10.1007/s11262-007-0187-8 18317917

[B20] Marinova-Petkova A. Franks J. Tenzin S. Dahal N. Dukpa K. Dorjee J.. (2016). Highly Pathogenic reassortant avian influenza A(H5N1) virus clade 2.3.2.1a in poultry, Bhutan. Emerg. Infect. Dis. 22, 2137–2141. 10.3201/eid2212.160611 27584733 PMC5189144

[B21] Nguyen T. H. Than V. T. Thanh H. D. Hung V. K. Nguyen D. T. Kim W. (2016). Intersubtype reassortments of H5N1 highly pathogenic avian influenza viruses isolated from Quail. PLoS ONE 11:e0149608. 10.1371/journal.pone.0149608 26900963 PMC4765837

[B22] Nobusawa E. Aoyama T. Kato H. Suzuki Y. Tateno Y. Nakajima K. (1991). Comparison of complete amino acid sequences and receptor-binding properties among 13 serotypes of hemagglutinins of influenza A viruses. Virology 182, 475–485. 10.1016/0042-6822(91)90588-3 2024485

[B23] Sakoda Y. Sugar S. Batchluun D. Erdene-Ochir T. O. Okamatsu M. Isoda N.. (2010). Characterization of H5N1 highly pathogenic avian influenza virus strains isolated from migratory waterfowl in Mongolia on the way back from the southern Asia to their northern territory. Virology 406, 88–94. 10.1016/j.virol.2010.07.007 20673942

[B24] Siboonnan N. Wiriyarat W. Boonarkart C. Chakritbudsabong W. Jongkaewwattana A. Puthavathana P.. (2013). A serine-to-asparagine mutation at position 314 of H5N1 avian influenza virus NP is a temperature-sensitive mutation that interferes with nuclear localization of NP. Arch. Virol. 158, 1151–1157. 10.1007/s00705-012-1595-1 23307364

[B25] Smith G. J. Vijaykrishna D. Ellis T. M. Dyrting K. C. Leung Y. H. Bahl J.. (2009). Characterization of avian influenza viruses A (H5N1) from wild birds, Hong Kong, 2004-2008. Emerg. Infect. Dis. 15, 402–407. 10.3201/eid1503.081190 19239752 PMC2666293

[B26] Song J. Feng H. Xu J. Zhao D. Shi J. Li Y.. (2011). The PA protein directly contributes to the virulence of H5N1 avian influenza viruses in domestic ducks. J. Virol. 85, 2180–2188. 10.1128/JVI.01975-10 21177821 PMC3067757

[B27] Subbarao K. Klimov A. Katz J. Regnery H. Lim W. Hall H.. (1998). Characterization of an avian influenza A (H5N1) virus isolated from a child with a fatal respiratory illness. Science 279, 393–396. 10.1126/science.279.5349.393 9430591

[B28] Sun H. Jiao P. Jia B. Xu C. Wei L. Shan F.. (2011). Pathogenicity in quails and mice of H5N1 highly pathogenic avian influenza viruses isolated from ducks. Vet. Microbiol. 152, 258–265. 10.1016/j.vetmic.2011.05.009 21665388

[B29] Suzuki H. Saito R. Masuda H. Oshitani H. Sato M. Sato I. (2003). Emergence of amantadine-resistant influenza A viruses: epidemiological study. J. Infect. Chemother. 9, 195–200. 10.1007/s10156-003-0262-6 14513385

[B30] Thakur A. K. Fezio W. L. (1981). A computer program for estimating LD50 and its confidence limits using modified Behrens-Reed-Muench cumulant method. Drug Chem. Toxicol. 4, 297–305. 10.3109/01480548109018136 7338208

[B31] Vijaykrishna D. Bahl J. Riley S. Duan L. Zhang J. X. Chen H.. (2008). Evolutionary dynamics and emergence of panzootic H5N1 influenza viruses. PLoS Pathog. 4:e1000161. 10.1371/journal.ppat.1000161 18818732 PMC2533123

[B32] Webster R. G. Bean W. J. Gorman O. T. Chambers T. M. Kawaoka Y.. (1992). Evolution and ecology of influenza A viruses. Microbiol. Rev. 56, 152–179. 1579108 10.1128/mr.56.1.152-179.1992 PMC372859

[B33] WHO (2011). Updated Unified Nomenclature System for the Highly Pathogenic H5N1 Avian Influenza Viruses. Available online at: http://www.who.int/influenza/gisrs_laboratory/h5n1_nomenclature/en. (Accessed).

[B34] WHO (2013). Cumulative Number of Confirmed Human Cases for Avian Influenza A(H5N1) Reported to WHO, 2003–2013. Available online at: http://www.who.int/influenza/human_animal_interface/EN_GIP_20130829CumulativeNumberH5N1cases.pdf (Accessed).

[B35] WHO (2015). Antigenic and genetic characteristics of zoonotic influenza viruses and development of candidate vaccine viruses for pandemic preparedness. Wkly. Epidemiol. Rec. 90, 561–571. Available online at: http://www.who.int/influenza/vaccines/virus/20170_zoonotic_vaccinevirusupdate.pdf 26477059

[B36] WHO (2017). Cumulative Number of Confirmed Human Cases for Avian Influenza A(H5N1) Reported to WHO, 2003–2017. Available online at: http://www.who.int/en/ (Accessed).

[B37] World Health Organization/World Organization for Animal Health/Food and Agriculture Organization (WHO/OIE/FAO) H5N1 Evolution Working Group, (2014). Revised and updated nomenclature for highly pathogenic avian influenza A (H5N1) viruses. Influenza Other Respir. Viruses 8, 384–388. 10.1111/irv.12230 24483237 PMC4181488

[B38] WHO (2012). Continued evolution of highly pathogenic avian influenza A (H5N1): updated nomenclature. Influenza Other Respir. Viruses 6, 1–5. 10.1111/j.1750-2659.2011.00298.x 22035148 PMC5074649

[B39] Yamaji R. Yamada S. Le M. Q. Ito M. Sakai-Tagawa Y. Kawaoka Y. (2015). Mammalian adaptive mutations of the PA protein of highly pathogenic avian H5N1 influenza virus. J. Virol. 89, 4117–4125. 10.1128/JVI.03532-14 25631084 PMC4442342

[B40] Yuan R. Cui J. Zhang S. Cao L. Liu X. Kang Y.. (2014). Pathogenicity and transmission of H5N1 avian influenza viruses in different birds. Vet. Microbiol. 168, 50–59. 10.1016/j.vetmic.2013.10.013 24268805

[B41] Zell R. Krumbholz A. Eitner A. Krieg R. Halbhuber K. J. Wutzler P. (2007). Prevalence of PB1-F2 of influenza A viruses. J. Gen. Virol. 88, 536–546. 10.1099/vir.0.82378-0 17251572

