# Supplementary material for: Phylogeny, Pathogenicity, and Transmission of H5N1 Avian Influenza Viruses in Chickens
Source: Front Cell Infect Microbiol. 2017 Jul 19;7:328. doi: 10.3389/fcimb.2017.00328 (PMC5515836; doi:10.3389/fcimb.2017.00328)
Supplement: Supplementary file 1 [file Table5.DOCX]

**Phylogeny, pathogenicity and transmission of H5N1 avian influenza viruses in chickens**

Jin Cui^1,2,3,4^, Nannan Qu^1,2,3,4^, Yang Guo^1,2,3,4^, Lan Cao^1, 4^, Siyu Wu^1,2,3,4^, Kun Mei^1,2,3,4^, Hailiang Song^1,2,3,4^, Yiliang Lu^5,6^, Zhifeng Qin^5^, Peirong Jiao^1,2,3,4*^ and Ming Liao^1,2,3,4*^

1 College of Veterinary Medicine, South China Agricultural University, Guangzhou, China

2 National and Regional Joint Engineering Laboratory for Medicament of Zoonosis Prevention and Control, Guangzhou, China

3 Key Laboratory of Zoonosis, Ministry of Agriculture, Guangzhou, China

4 Guangzhou Center for Disease Control and Prevention, Guangzhou, China

5 Animal & plant inspection and quarantine technology center of Shenzhen entry-exit inspection and quarantine bureau of China, Shenzhen, China

6 College of life sciences and oceanography, Shenzhen university, Shenzhen, China

* Peirong Jiao and Ming Liao are the Corresponding author:

Dr. Peirong Jiao, College of Veterinary Medicine, South China Agricultural University, 483 Wushan Road, Tianhe District, Guangzhou, 510642, China.

Tel: (+86) 020-85283309; Fax: (+86) 020-85280234; E-mail: prjiao@scau.edu.cn

Dr. Ming Liao, College of Veterinary Medicine, South China Agricultural University, 483 Wushan Road, Tianhe District, Guangzhou, 510642, China.

Tel: (+86) 020-85285282; Fax: (+86) 020-85285582; E-mail: mliao@scau.edu.cn

**SUPPLEMENTARY TABLE**

| Supplementary Table 5. NCBI GenBank accession number of the sequence in the phylogenetic tree | | |
| --- | --- | --- |
| Accession | Segment | Virus name |
| CY098292 | 4 (HA) | Influenza A virus (A/tundra swan/Mongolia/1T/2010(H5N1)) |
| KC871423 | 7 (MP) | Influenza A virus (A/tundra swan/Mongolia/1T2/2010(H5N1)) |
| KC871424 | 8 (NS) | Influenza A virus (A/tundra swan/Mongolia/1T2/2010(H5N1)) |
| KC886648 | 5 (NP) | Influenza A virus (A/tundra swan/Mongolia/1T2/2010(H5N1)) |
| KC986298 | 1 (PB2) | Influenza A virus (A/tundra swan/Mongolia/1T2/2010(H5N1)) |
| KF501051 | 4 (HA) | Influenza A virus (A/tundra swan/Mongolia/1T2/2010(H5N1)) |
| KF454780 | 3 (PA) | Influenza A virus (A/tundra swan/Mongolia/1T2/2010(H5N1)) |
| AB523764 | 1 (PB2) | Influenza A virus (A/bar-headed goose/Mongolia/X53/2009(H5N1)) |
| AB523765 | 2 (PB1) | Influenza A virus (A/bar-headed goose/Mongolia/X53/2009(H5N1)) |
| AB523766 | 3 (PA) | Influenza A virus (A/bar-headed goose/Mongolia/X53/2009(H5N1)) |
| AB523767 | 4 (HA) | Influenza A virus (A/bar-headed goose/Mongolia/X53/2009(H5N1)) |
| AB523768 | 5 (NP) | Influenza A virus (A/bar-headed goose/Mongolia/X53/2009(H5N1)) |
| AB523769 | 6 (NA) | Influenza A virus (A/bar-headed goose/Mongolia/X53/2009(H5N1)) |
| AB523770 | 7 (MP) | Influenza A virus (A/bar-headed goose/Mongolia/X53/2009(H5N1)) |
| AB523771 | 8 (NS) | Influenza A virus (A/bar-headed goose/Mongolia/X53/2009(H5N1)) |
| CY098291 | 4 (HA) | Influenza A virus (A/herring gull/Mongolia/833T/2009(H5N1)) |
| KC871455 | 7 (MP) | Influenza A virus (A/herring gull/Mongolia/833T2/2009(H5N1)) |
| KC871456 | 8 (NS) | Influenza A virus (A/herring gull/Mongolia/833T2/2009(H5N1)) |
| KC986315 | 1 (PB2) | Influenza A virus (A/herring gull/Mongolia/833T2/2009(H5N1)) |
| KC886665 | 5 (NP) | Influenza A virus (A/herring gull/Mongolia/833T2/2009(H5N1)) |
| KF454797 | 3 (PA) | Influenza A virus (A/herring gull/Mongolia/833T2/2009(H5N1)) |
| KC992208 | 2 (PB1) | Influenza A virus (A/herring gull/Mongolia/833T2/2009(H5N1)) |
| KF667732 | 6 (NA) | Influenza A virus (A/herring gull/Mongolia/833T2/2009(H5N1)) |
| KF501065 | 4 (HA) | Influenza A virus (A/herring gull/Mongolia/833T2/2009(H5N1)) |
| CY036202 | 1 (PB2) | Influenza A virus (A/little egret/Hong Kong/8863/2007(H5N1)) |
| CY036203 | 2 (PB1) | Influenza A virus (A/little egret/Hong Kong/8863/2007(H5N1)) |
| CY036204 | 3 (PA) | Influenza A virus (A/little egret/Hong Kong/8863/2007(H5N1)) |
| CY036205 | 4 (HA) | Influenza A virus (A/little egret/Hong Kong/8863/2007(H5N1)) |
| CY036206 | 5 (NP) | Influenza A virus (A/little egret/Hong Kong/8863/2007(H5N1)) |
| CY036207 | 6 (NA) | Influenza A virus (A/little egret/Hong Kong/8863/2007(H5N1)) |
| CY036208 | 7 (MP) | Influenza A virus (A/little egret/Hong Kong/8863/2007(H5N1)) |
| CY036209 | 8 (NS) | Influenza A virus (A/little egret/Hong Kong/8863/2007(H5N1)) |
| CY036226 | 1 (PB2) | Influenza A virus (A/black-crowned night heron/Hong Kong/659/2008(H5N1)) |
| CY036227 | 2 (PB1) | Influenza A virus (A/black-crowned night heron/Hong Kong/659/2008(H5N1)) |
| CY036228 | 3 (PA) | Influenza A virus (A/black-crowned night heron/Hong Kong/659/2008(H5N1)) |
| CY036229 | 4 (HA) | Influenza A virus (A/black-crowned night heron/Hong Kong/659/2008(H5N1)) |
| CY036230 | 5 (NP) | Influenza A virus (A/black-crowned night heron/Hong Kong/659/2008(H5N1)) |
| CY036231 | 6 (NA) | Influenza A virus (A/black-crowned night heron/Hong Kong/659/2008(H5N1)) |
| CY036232 | 7 (MP) | Influenza A virus (A/black-crowned night heron/Hong Kong/659/2008(H5N1)) |
| CY036233 | 8 (NS) | Influenza A virus (A/black-crowned night heron/Hong Kong/659/2008(H5N1)) |
| CY036218 | 1 (PB2) | Influenza A virus (A/common buzzard/Hong Kong/9213/2007(H5N1)) |
| CY036219 | 2 (PB1) | Influenza A virus (A/common buzzard/Hong Kong/9213/2007(H5N1)) |
| CY036220 | 3 (PA) | Influenza A virus (A/common buzzard/Hong Kong/9213/2007(H5N1)) |
| CY036221 | 4 (HA) | Influenza A virus (A/common buzzard/Hong Kong/9213/2007(H5N1)) |
| CY036222 | 5 (NP) | Influenza A virus (A/common buzzard/Hong Kong/9213/2007(H5N1)) |
| CY036223 | 6 (NA) | Influenza A virus (A/common buzzard/Hong Kong/9213/2007(H5N1)) |
| CY036224 | 7 (MP) | Influenza A virus (A/common buzzard/Hong Kong/9213/2007(H5N1)) |
| CY036225 | 8 (NS) | Influenza A virus (A/common buzzard/Hong Kong/9213/2007(H5N1)) |
| CY036250 | 1 (PB2) | Influenza A virus (A/magpie robin/Hong Kong/1097/2008(H5N1)) |
| CY036251 | 2 (PB1) | Influenza A virus (A/magpie robin/Hong Kong/1097/2008(H5N1)) |
| CY036252 | 3 (PA) | Influenza A virus (A/magpie robin/Hong Kong/1097/2008(H5N1)) |
| CY036253 | 4 (HA) | Influenza A virus (A/magpie robin/Hong Kong/1097/2008(H5N1)) |
| CY036254 | 5 (NP) | Influenza A virus (A/magpie robin/Hong Kong/1097/2008(H5N1)) |
| CY036255 | 6 (NA) | Influenza A virus (A/magpie robin/Hong Kong/1097/2008(H5N1)) |
| CY036257 | 8 (NS) | Influenza A virus (A/magpie robin/Hong Kong/1097/2008(H5N1)) |
| CY036256 | 7 (MP) | Influenza A virus (A/magpie robin/Hong Kong/1097/2008(H5N1)) |
| CY036258 | 1 (PB2) | Influenza A virus (A/magpie robin/Hong Kong/1897/2008(H5N1)) |
| CY036259 | 2 (PB1) | Influenza A virus (A/magpie robin/Hong Kong/1897/2008(H5N1)) |
| CY036260 | 3 (PA) | Influenza A virus (A/magpie robin/Hong Kong/1897/2008(H5N1)) |
| CY036261 | 4 (HA) | Influenza A virus (A/magpie robin/Hong Kong/1897/2008(H5N1)) |
| CY036262 | 5 (NP) | Influenza A virus (A/magpie robin/Hong Kong/1897/2008(H5N1)) |
| CY036263 | 6 (NA) | Influenza A virus (A/magpie robin/Hong Kong/1897/2008(H5N1)) |
| CY036264 | 7 (MP) | Influenza A virus (A/magpie robin/Hong Kong/1897/2008(H5N1)) |
| CY036265 | 8 (NS) | Influenza A virus (A/magpie robin/Hong Kong/1897/2008(H5N1)) |
| CY036210 | 1 (PB2) | Influenza A virus (A/grey heron/Hong Kong/3088/2007(H5N1)) |
| CY036211 | 2 (PB1) | Influenza A virus (A/grey heron/Hong Kong/3088/2007(H5N1)) |
| CY036212 | 3 (PA) | Influenza A virus (A/grey heron/Hong Kong/3088/2007(H5N1)) |
| CY036213 | 4 (HA) | Influenza A virus (A/grey heron/Hong Kong/3088/2007(H5N1)) |
| CY036214 | 5 (NP) | Influenza A virus (A/grey heron/Hong Kong/3088/2007(H5N1)) |
| CY036215 | 6 (NA) | Influenza A virus (A/grey heron/Hong Kong/3088/2007(H5N1)) |
| CY036216 | 7 (MP) | Influenza A virus (A/grey heron/Hong Kong/3088/2007(H5N1)) |
| CY036217 | 8 (NS) | Influenza A virus (A/grey heron/Hong Kong/3088/2007(H5N1)) |
| KC357320 | 4 (HA) | Influenza A virus (A/barn swallow/Hong Kong/1161/2010(H5N1)) |
| KC436130 | 7 (MP) | Influenza A virus (A/barn swallow/Hong Kong/1161/2010(H5N1)) |
| KF735641 | 1 (PB2) | Influenza A virus (A/barn swallow/Hong Kong/1161/2010(H5N1)) |
| KF735642 | 2 (PB1) | Influenza A virus (A/barn swallow/Hong Kong/1161/2010(H5N1)) |
| KF735643 | 3 (PA) | Influenza A virus (A/barn swallow/Hong Kong/1161/2010(H5N1)) |
| KF735644 | 5 (NP) | Influenza A virus (A/barn swallow/Hong Kong/1161/2010(H5N1)) |
| KF735645 | 6 (NA) | Influenza A virus (A/barn swallow/Hong Kong/1161/2010(H5N1)) |
| KF735646 | 8 (NS) | Influenza A virus (A/barn swallow/Hong Kong/1161/2010(H5N1)) |
| AY651349 | 4 (HA) | Influenza A virus (A/Ck/HK/YU22/2002(H5N1)) |
| AY651568 | 8 (NS) | Influenza A virus (A/Ck/HK/YU22/2002(H5N1)) |
| AY651403 | 7 (MP) | Influenza A virus (A/Ck/HK/YU22/2002(H5N1)) |
| AY651461 | 6 (NA) | Influenza A virus (A/Ck/HK/YU22/2002(H5N1)) |
| AY651514 | 5 (NP) | Influenza A virus (A/Ck/HK/YU22/2002(H5N1)) |
| AY651626 | 3 (PA) | Influenza A virus (A/Ck/HK/YU22/2002(H5N1)) |
| AY651680 | 2 (PB1) | Influenza A virus (A/Ck/HK/YU22/2002(H5N1)) |
| AY651734 | 1 (PB2) | Influenza A virus (A/Ck/HK/YU22/2002(H5N1)) |
| AY576375 | 8 (NS) | Influenza A virus (A/Ck/HK/61.9/02 (H5N1)) |
| AY576387 | 1 (PB2) | Influenza A virus (A/Ck/HK/61.9/02 (H5N1)) |
| AY576399 | 2 (PB1) | Influenza A virus (A/Ck/HK/61.9/02 (H5N1)) |
| AY576411 | 3 (PA) | Influenza A virus (A/Ck/HK/61.9/02 (H5N1)) |
| AY575876 | 4 (HA) | Influenza A virus (A/Ck/HK/61.9/02 (H5N1)) |
| AY575888 | 6 (NA) | Influenza A virus (A/Ck/HK/61.9/02 (H5N1)) |
| AY575900 | 7 (MP) | Influenza A virus (A/Ck/HK/61.9/02 (H5N1)) |
| AY575912 | 5 (NP) | Influenza A virus (A/Ck/HK/61.9/02 (H5N1)) |
| AF509077 | 8 (NS) | Influenza A virus (A/Chicken/Hong Kong/822.1/01 (H5N1)) |
| AF509026 | 4 (HA) | Influenza A virus (A/Chicken/Hong Kong/822.1/01 (H5N1)) |
| AF509102 | 6 (NA) | Influenza A virus (A/Chicken/Hong Kong/822.1/01 (H5N1)) |
| AF509128 | 5 (NP) | Influenza A virus (A/Chicken/Hong Kong/822.1/01 (H5N1)) |
| AF509154 | 1 (PB2) | Influenza A virus (A/Chicken/Hong Kong/822.1/01 (H5N1)) |
| AF509180 | 2 (PB1) | Influenza A virus (A/Chicken/Hong Kong/822.1/01 (H5N1)) |
| AF509206 | 3 (PA) | Influenza A virus (A/Chicken/Hong Kong/822.1/01 (H5N1)) |
| AF509017 | 4 (HA) | Influenza A virus (A/chicken/Hong Kong/YU562/2001(H5N1)) |
| AF509093 | 6 (NA) | Influenza A virus (A/chicken/Hong Kong/YU562/2001(H5N1)) |
| AF509118 | 5 (NP) | Influenza A virus (A/chicken/Hong Kong/YU562/2001(H5N1)) |
| AF509144 | 1 (PB2) | Influenza A virus (A/chicken/Hong Kong/YU562/2001(H5N1)) |
| AF509170 | 2 (PB1) | Influenza A virus (A/chicken/Hong Kong/YU562/2001(H5N1)) |
| AF509196 | 3 (PA) | Influenza A virus (A/chicken/Hong Kong/YU562/2001(H5N1)) |
| AY221529 | 4 (HA) | Influenza A virus (A/chicken/Hong Kong/YU562/2001(H5N1)) |
| AF509016 | 4 (HA) | Influenza A virus (A/Chicken/Hong Kong/FY77/01 (H5N1)) |
| AF509040 | 7 (MP) | Influenza A virus (A/Chicken/Hong Kong/FY77/01 (H5N1)) |
| AF509066 | 8 (NS) | Influenza A virus (A/Chicken/Hong Kong/FY77/01 (H5N1)) |
| AF509092 | 6 (NA) | Influenza A virus (A/Chicken/Hong Kong/FY77/01 (H5N1)) |
| AF509117 | 5 (NP) | Influenza A virus (A/Chicken/Hong Kong/FY77/01 (H5N1)) |
| AF509143 | 1 (PB2) | Influenza A virus (A/Chicken/Hong Kong/FY77/01 (H5N1)) |
| AF509169 | 2 (PB1) | Influenza A virus (A/Chicken/Hong Kong/FY77/01 (H5N1)) |
| AF509195 | 3 (PA) | Influenza A virus (A/Chicken/Hong Kong/FY77/01 (H5N1)) |
| JN795913 | 4 (HA) | Influenza A virus (A/crow/Bangladesh/11rs1984-11/2011(H5N1)) |
| HM172081 | 4 (HA) | Influenza A virus (A/chicken/Hebei/A-8/2009(H5N1)) |
| HM172137 | 7 (MP) | Influenza A virus (A/chicken/Hebei/A-8/2009(H5N1)) |
| HM172197 | 6 (NA) | Influenza A virus (A/chicken/Hebei/A-8/2009(H5N1)) |
| HM172217 | 5 (NP) | Influenza A virus (A/chicken/Hebei/A-8/2009(H5N1)) |
| HM172271 | 8 (NS) | Influenza A virus (A/chicken/Hebei/A-8/2009(H5N1)) |
| HM172324 | 3 (PA) | Influenza A virus (A/chicken/Hebei/A-8/2009(H5N1)) |
| HM172366 | 2 (PB1) | Influenza A virus (A/chicken/Hebei/A-8/2009(H5N1)) |
| HM172411 | 1 (PB2) | Influenza A virus (A/chicken/Hebei/A-8/2009(H5N1)) |
| DQ351875 | 2 (PB1) | Influenza A virus (A/chicken/Hebei/326/2005(H5N1)) |
| DQ351871 | 1 (PB2) | Influenza A virus (A/chicken/Hebei/326/2005(H5N1)) |
| DQ351868 | 3 (PA) | Influenza A virus (A/chicken/Hebei/326/2005(H5N1)) |
| DQ349118 | 6 (NA) | Influenza A virus (A/chicken/Hebei/326/2005(H5N1)) |
| DQ351859 | 7 (MP) | Influenza A virus (A/chicken/Hebei/326/2005(H5N1)) |
| DQ351865 | 5 (NP) | Influenza A virus (A/chicken/Hebei/326/2005(H5N1)) |
| DQ351862 | 8 (NS) | Influenza A virus (A/chicken/Hebei/326/2005(H5N1)) |
| DQ343150 | 4 (HA) | Influenza A virus (A/chicken/Hebei/326/2005(H5N1)) |
| AY741213 | 4 (HA) | Influenza A virus (A/blackbird/Hunan/1/2004(H5N1)) |
| AY741214 | 6 (NA) | Influenza A virus (A/blackbird/Hunan/1/2004(H5N1)) |
| DQ835774 | 7 (MP) | Influenza A virus (A/blackbird/Hunan/1/2004(H5N1)) |
| DQ835779 | 5 (NP) | Influenza A virus (A/blackbird/Hunan/1/2004(H5N1)) |
| DQ835790 | 3 (PA) | Influenza A virus (A/blackbird/Hunan/1/2004(H5N1)) |
| DQ835793 | 2 (PB1) | Influenza A virus (A/blackbird/Hunan/1/2004(H5N1)) |
| DQ835805 | 1 (PB2) | Influenza A virus (A/blackbird/Hunan/1/2004(H5N1)) |
| DQ835811 | 8 (NS) | Influenza A virus (A/blackbird/Hunan/1/2004(H5N1)) |
| GU182155 | 1 (PB2) | Influenza A virus (A/chicken/Hunan/8/2008(H5N1)) |
| GU182156 | 2 (PB1) | Influenza A virus (A/chicken/Hunan/8/2008(H5N1)) |
| GU182157 | 3 (PA) | Influenza A virus (A/chicken/Hunan/8/2008(H5N1)) |
| GU182158 | 4 (HA) | Influenza A virus (A/chicken/Hunan/8/2008(H5N1)) |
| GU182159 | 5 (NP) | Influenza A virus (A/chicken/Hunan/8/2008(H5N1)) |
| GU182160 | 6 (NA) | Influenza A virus (A/chicken/Hunan/8/2008(H5N1)) |
| GU182162 | 7 (MP) | Influenza A virus (A/chicken/Hunan/8/2008(H5N1)) |
| GU182161 | 8 (NS) | Influenza A virus (A/chicken/Hunan/8/2008(H5N1)) |
| EU430496 | 4 (HA) | Influenza A virus (A/domestic green-winged teal/Hunan/67/2005(H5N1)) |
| EU430497 | 6 (NA) | Influenza A virus (A/domestic green-winged teal/Hunan/67/2005(H5N1)) |
| EU430498 | 5 (NP) | Influenza A virus (A/domestic green-winged teal/Hunan/67/2005(H5N1)) |
| EU430499 | 1 (PB2) | Influenza A virus (A/domestic green-winged teal/Hunan/67/2005(H5N1)) |
| EU430500 | 2 (PB1) | Influenza A virus (A/domestic green-winged teal/Hunan/67/2005(H5N1)) |
| EU430501 | 3 (PA) | Influenza A virus (A/domestic green-winged teal/Hunan/67/2005(H5N1)) |
| EU430502 | 7 (MP) | Influenza A virus (A/domestic green-winged teal/Hunan/67/2005(H5N1)) |
| EU430503 | 8 (NS) | Influenza A virus (A/domestic green-winged teal/Hunan/67/2005(H5N1)) |
| DQ321231 | 3 (PA) | Influenza A virus (A/duck/Hunan/127/2005(H5N1)) |
| DQ321099 | 5 (NP) | Influenza A virus (A/duck/Hunan/127/2005(H5N1)) |
| DQ321297 | 2 (PB1) | Influenza A virus (A/duck/Hunan/127/2005(H5N1)) |
| DQ321165 | 8 (NS) | Influenza A virus (A/duck/Hunan/127/2005(H5N1)) |
| DQ320968 | 7 (MP) | Influenza A virus (A/duck/Hunan/127/2005(H5N1)) |
| DQ321034 | 6 (NA) | Influenza A virus (A/duck/Hunan/127/2005(H5N1)) |
| DQ320836 | 1 (PB2) | Influenza A virus (A/duck/Hunan/127/2005(H5N1)) |
| DQ320902 | 4 (HA) | Influenza A virus (A/duck/Hunan/127/2005(H5N1)) |
| DQ320903 | 4 (HA) | Influenza A virus (A/duck/Hunan/139/2005(H5N1)) |
| DQ320837 | 1 (PB2) | Influenza A virus (A/duck/Hunan/139/2005(H5N1)) |
| DQ321035 | 6 (NA) | Influenza A virus (A/duck/Hunan/139/2005(H5N1)) |
| DQ320969 | 7 (MP) | Influenza A virus (A/duck/Hunan/139/2005(H5N1)) |
| DQ321166 | 8 (NS) | Influenza A virus (A/duck/Hunan/139/2005(H5N1)) |
| DQ321298 | 2 (PB1) | Influenza A virus (A/duck/Hunan/139/2005(H5N1)) |
| DQ321100 | 5 (NP) | Influenza A virus (A/duck/Hunan/139/2005(H5N1)) |
| DQ321232 | 3 (PA) | Influenza A virus (A/duck/Hunan/139/2005(H5N1)) |
| DQ320905 | 4 (HA) | Influenza A virus (A/duck/Hunan/152/2005(H5N1)) |
| DQ320839 | 1 (PB2) | Influenza A virus (A/duck/Hunan/152/2005(H5N1)) |
| DQ321037 | 6 (NA) | Influenza A virus (A/duck/Hunan/152/2005(H5N1)) |
| DQ320971 | 7 (MP) | Influenza A virus (A/duck/Hunan/152/2005(H5N1)) |
| DQ321168 | 8 (NS) | Influenza A virus (A/duck/Hunan/152/2005(H5N1)) |
| DQ321300 | 2 (PB1) | Influenza A virus (A/duck/Hunan/152/2005(H5N1)) |
| DQ321102 | 5 (NP) | Influenza A virus (A/duck/Hunan/152/2005(H5N1)) |
| DQ321234 | 3 (PA) | Influenza A virus (A/duck/Hunan/152/2005(H5N1)) |
| DQ321142 | 8 (NS) | Influenza A virus (A/duck/Guangxi/668/2004(H5N1)) |
| DQ321274 | 2 (PB1) | Influenza A virus (A/duck/Guangxi/668/2004(H5N1)) |
| DQ321208 | 3 (PA) | Influenza A virus (A/duck/Guangxi/668/2004(H5N1)) |
| DQ320813 | 1 (PB2) | Influenza A virus (A/duck/Guangxi/668/2004(H5N1)) |
| DQ320879 | 4 (HA) | Influenza A virus (A/duck/Guangxi/668/2004(H5N1)) |
| DQ320945 | 7 (MP) | Influenza A virus (A/duck/Guangxi/668/2004(H5N1)) |
| DQ321076 | 5 (NP) | Influenza A virus (A/duck/Guangxi/668/2004(H5N1)) |
| DQ321012 | 6 (NA) | Influenza A virus (A/goose/Guangxi/914/2004(H5N1)) |
| DQ320946 | 7 (MP) | Influenza A virus (A/goose/Guangxi/914/2004(H5N1)) |
| DQ321077 | 5 (NP) | Influenza A virus (A/goose/Guangxi/914/2004(H5N1)) |
| DQ320880 | 4 (HA) | Influenza A virus (A/goose/Guangxi/914/2004(H5N1)) |
| DQ320814 | 1 (PB2) | Influenza A virus (A/goose/Guangxi/914/2004(H5N1)) |
| DQ321209 | 3 (PA) | Influenza A virus (A/goose/Guangxi/914/2004(H5N1)) |
| DQ321275 | 2 (PB1) | Influenza A virus (A/goose/Guangxi/914/2004(H5N1)) |
| DQ321143 | 8 (NS) | Influenza A virus (A/goose/Guangxi/914/2004(H5N1)) |
| KT762436 | 1 (PB2) | Influenza A virus (A/chicken/Guangxi/S2039/2009(H5N1)) |
| KT762437 | 2 (PB1) | Influenza A virus (A/chicken/Guangxi/S2039/2009(H5N1)) |
| KT762438 | 3 (PA) | Influenza A virus (A/chicken/Guangxi/S2039/2009(H5N1)) |
| KT762439 | 4 (HA) | Influenza A virus (A/chicken/Guangxi/S2039/2009(H5N1)) |
| KT762440 | 5 (NP) | Influenza A virus (A/chicken/Guangxi/S2039/2009(H5N1)) |
| KT762441 | 6 (NA) | Influenza A virus (A/chicken/Guangxi/S2039/2009(H5N1)) |
| KT762442 | 7 (MP) | Influenza A virus (A/chicken/Guangxi/S2039/2009(H5N1)) |
| KT762443 | 8 (NS) | Influenza A virus (A/chicken/Guangxi/S2039/2009(H5N1)) |
| KP097846 | 1 (PB2) | Influenza A virus (A/duck/Vietnam/NCVD-A672/2011(H5N1)) |
| KP097867 | 2 (PB1) | Influenza A virus (A/duck/Vietnam/NCVD-A672/2011(H5N1)) |
| KP097888 | 3 (PA) | Influenza A virus (A/duck/Vietnam/NCVD-A672/2011(H5N1)) |
| KP097923 | 4 (HA) | Influenza A virus (A/duck/Vietnam/NCVD-A672/2011(H5N1)) |
| KP097944 | 5 (NP) | Influenza A virus (A/duck/Vietnam/NCVD-A672/2011(H5N1)) |
| KP097979 | 6 (NA) | Influenza A virus (A/duck/Vietnam/NCVD-A672/2011(H5N1)) |
| KP098000 | 7 (MP) | Influenza A virus (A/duck/Vietnam/NCVD-A672/2011(H5N1)) |
| KP098021 | 8 (NS) | Influenza A virus (A/duck/Vietnam/NCVD-A672/2011(H5N1)) |
| AB741563 | 1 (PB2) | Influenza A virus (A/muscovy duck/Vietnam/LBM66/2011 (H5N1)) |
| AB741564 | 2 (PB1) | Influenza A virus (A/muscovy duck/Vietnam/LBM66/2011 (H5N1)) |
| AB741565 | 3 (PA) | Influenza A virus (A/muscovy duck/Vietnam/LBM66/2011 (H5N1)) |
| AB741566 | 4 (HA) | Influenza A virus (A/muscovy duck/Vietnam/LBM66/2011 (H5N1)) |
| AB741567 | 5 (NP) | Influenza A virus (A/muscovy duck/Vietnam/LBM66/2011 (H5N1)) |
| AB741568 | 6 (NA) | Influenza A virus (A/muscovy duck/Vietnam/LBM66/2011 (H5N1)) |
| AB741569 | 7 (MP) | Influenza A virus (A/muscovy duck/Vietnam/LBM66/2011 (H5N1)) |
| AB741570 | 8 (NS) | Influenza A virus (A/muscovy duck/Vietnam/LBM66/2011 (H5N1)) |
| KP097828 | 1 (PB2) | Influenza A virus (A/duck/Vietnam/NCVD-KA25/2011(H5N1)) |
| KP097849 | 2 (PB1) | Influenza A virus (A/duck/Vietnam/NCVD-KA25/2011(H5N1)) |
| KP097870 | 3 (PA) | Influenza A virus (A/duck/Vietnam/NCVD-KA25/2011(H5N1)) |
| KP097891 | 4 (HA) | Influenza A virus (A/duck/Vietnam/NCVD-KA25/2011(H5N1)) |
| KP097926 | 5 (NP) | Influenza A virus (A/duck/Vietnam/NCVD-KA25/2011(H5N1)) |
| KP097947 | 6 (NA) | Influenza A virus (A/duck/Vietnam/NCVD-KA25/2011(H5N1)) |
| KP097982 | 7 (MP) | Influenza A virus (A/duck/Vietnam/NCVD-KA25/2011(H5N1)) |
| KP098003 | 8 (NS) | Influenza A virus (A/duck/Vietnam/NCVD-KA25/2011(H5N1)) |
| DQ997401 | 3 (PA) | Influenza A virus (A/goose/Fujian/bb/2003(H5N1)) |
| DQ997402 | 7 (MP) | Influenza A virus (A/goose/Fujian/bb/2003(H5N1)) |
| DQ997403 | 2 (PB1) | Influenza A virus (A/goose/Fujian/bb/2003(H5N1)) |
| DQ997404 | 1 (PB2) | Influenza A virus (A/goose/Fujian/bb/2003(H5N1)) |
| DQ997405 | 4 (HA) | Influenza A virus (A/goose/Fujian/bb/2003(H5N1)) |
| DQ997406 | 6 (NA) | Influenza A virus (A/goose/Fujian/bb/2003(H5N1)) |
| DQ997407 | 5 (NP) | Influenza A virus (A/goose/Fujian/bb/2003(H5N1)) |
| DQ997408 | 8 (NS) | Influenza A virus (A/goose/Fujian/bb/2003(H5N1)) |
| JX534594 | 1 (PB2) | Influenza A virus (A/wild duck/Fujian/2/2011(H5N1)) |
| JX534595 | 2 (PB1) | Influenza A virus (A/wild duck/Fujian/2/2011(H5N1)) |
| JX534596 | 3 (PA) | Influenza A virus (A/wild duck/Fujian/2/2011(H5N1)) |
| JX534597 | 4 (HA) | Influenza A virus (A/wild duck/Fujian/2/2011(H5N1)) |
| JX534598 | 5 (NP) | Influenza A virus (A/wild duck/Fujian/2/2011(H5N1)) |
| JX534599 | 6 (NA) | Influenza A virus (A/wild duck/Fujian/2/2011(H5N1)) |
| JX534600 | 7 (MP) | Influenza A virus (A/wild duck/Fujian/2/2011(H5N1)) |
| JX534601 | 8 (NS) | Influenza A virus (A/wild duck/Fujian/2/2011(H5N1)) |
| HM172182 | 6 (NA) | Influenza A virus (A/duck/Anhui/1/06(H5N1)) |
| HM172133 | 7 (MP) | Influenza A virus (A/duck/Anhui/1/06(H5N1)) |
| HM172401 | 2 (PB1) | Influenza A virus (A/duck/Anhui/1/06(H5N1)) |
| HM172409 | 1 (PB2) | Influenza A virus (A/duck/Anhui/1/06(H5N1)) |
| HM172115 | 4 (HA) | Influenza A virus (A/duck/Anhui/1/06(H5N1)) |
| HM172356 | 3 (PA) | Influenza A virus (A/duck/Anhui/1/06(H5N1)) |
| HM172304 | 8 (NS) | Influenza A virus (A/duck/Anhui/1/06(H5N1)) |
| HM172238 | 5 (NP) | Influenza A virus (A/duck/Anhui/1/06(H5N1)) |
| DQ992676 | 1 (PB2) | Influenza A virus (A/duck/Guiyang/3009/2005(H5N1)) |
| DQ992754 | 4 (HA) | Influenza A virus (A/duck/Guiyang/3009/2005(H5N1)) |
| EF124153 | 7 (MP) | Influenza A virus (A/duck/Guiyang/3009/2005(H5N1)) |
| EF124002 | 2 (PB1) | Influenza A virus (A/duck/Guiyang/3009/2005(H5N1)) |
| EF124304 | 6 (NA) | Influenza A virus (A/duck/Guiyang/3009/2005(H5N1)) |
| EF124606 | 8 (NS) | Influenza A virus (A/duck/Guiyang/3009/2005(H5N1)) |
| EF124455 | 5 (NP) | Influenza A virus (A/duck/Guiyang/3009/2005(H5N1)) |
| EF124757 | 3 (PA) | Influenza A virus (A/duck/Guiyang/3009/2005(H5N1)) |
| DQ992756 | 4 (HA) | Influenza A virus (A/duck/Guiyang/3242/2005(H5N1)) |
| DQ992678 | 1 (PB2) | Influenza A virus (A/duck/Guiyang/3242/2005(H5N1)) |
| EF124004 | 2 (PB1) | Influenza A virus (A/duck/Guiyang/3242/2005(H5N1)) |
| EF124155 | 7 (MP) | Influenza A virus (A/duck/Guiyang/3242/2005(H5N1)) |
| EF124457 | 5 (NP) | Influenza A virus (A/duck/Guiyang/3242/2005(H5N1)) |
| EF124608 | 8 (NS) | Influenza A virus (A/duck/Guiyang/3242/2005(H5N1)) |
| EF124306 | 6 (NA) | Influenza A virus (A/duck/Guiyang/3242/2005(H5N1)) |
| EF124759 | 3 (PA) | Influenza A virus (A/duck/Guiyang/3242/2005(H5N1)) |
| EF124660 | 3 (PA) | Influenza A virus (A/goose/Guiyang/337/2006(H5N1)) |
| DQ992765 | 4 (HA) | Influenza A virus (A/goose/Guiyang/337/2006(H5N1)) |
| DQ992579 | 1 (PB2) | Influenza A virus (A/goose/Guiyang/337/2006(H5N1)) |
| EF124207 | 6 (NA) | Influenza A virus (A/goose/Guiyang/337/2006(H5N1)) |
| EF124358 | 5 (NP) | Influenza A virus (A/goose/Guiyang/337/2006(H5N1)) |
| EF124509 | 8 (NS) | Influenza A virus (A/goose/Guiyang/337/2006(H5N1)) |
| EF124056 | 7 (MP) | Influenza A virus (A/goose/Guiyang/337/2006(H5N1)) |
| EF123905 | 2 (PB1) | Influenza A virus (A/goose/Guiyang/337/2006(H5N1)) |
| DQ992679 | 1 (PB2) | Influenza A virus (A/goose/Guiyang/3422/2005(H5N1)) |
| DQ992757 | 4 (HA) | Influenza A virus (A/goose/Guiyang/3422/2005(H5N1)) |
| EF124005 | 2 (PB1) | Influenza A virus (A/goose/Guiyang/3422/2005(H5N1)) |
| EF124156 | 7 (MP) | Influenza A virus (A/goose/Guiyang/3422/2005(H5N1)) |
| EF124458 | 5 (NP) | Influenza A virus (A/goose/Guiyang/3422/2005(H5N1)) |
| EF124609 | 8 (NS) | Influenza A virus (A/goose/Guiyang/3422/2005(H5N1)) |
| EF124307 | 6 (NA) | Influenza A virus (A/goose/Guiyang/3422/2005(H5N1)) |
| EF124760 | 3 (PA) | Influenza A virus (A/goose/Guiyang/3422/2005(H5N1)) |
| EF124758 | 3 (PA) | Influenza A virus (A/chicken/Guiyang/3055/2005(H5N1)) |
| EF124607 | 8 (NS) | Influenza A virus (A/chicken/Guiyang/3055/2005(H5N1)) |
| EF124456 | 5 (NP) | Influenza A virus (A/chicken/Guiyang/3055/2005(H5N1)) |
| EF124305 | 6 (NA) | Influenza A virus (A/chicken/Guiyang/3055/2005(H5N1)) |
| EF124154 | 7 (MP) | Influenza A virus (A/chicken/Guiyang/3055/2005(H5N1)) |
| EF124003 | 2 (PB1) | Influenza A virus (A/chicken/Guiyang/3055/2005(H5N1)) |
| DQ992677 | 1 (PB2) | Influenza A virus (A/chicken/Guiyang/3055/2005(H5N1)) |
| DQ992755 | 4 (HA) | Influenza A virus (A/chicken/Guiyang/3055/2005(H5N1)) |
| EF619973 | 6 (NA) | Influenza A virus (A/turkey/Turkey/1/2005(H5N1)) |
| EF619974 | 8 (NS) | Influenza A virus (A/turkey/Turkey/1/2005(H5N1)) |
| EF619975 | 1 (PB2) | Influenza A virus (A/turkey/Turkey/1/2005(H5N1)) |
| EF619976 | 2 (PB1) | Influenza A virus (A/turkey/Turkey/1/2005(H5N1)) |
| EF619977 | 5 (NP) | Influenza A virus (A/turkey/Turkey/1/2005(H5N1)) |
| EF619978 | 7 (MP) | Influenza A virus (A/turkey/Turkey/1/2005(H5N1)) |
| EF619979 | 3 (PA) | Influenza A virus (A/turkey/Turkey/1/2005(H5N1)) |
| EF619980 | 4 (HA) | Influenza A virus (A/turkey/Turkey/1/2005(H5N1)) |
| DQ095764 | 1 (PB2) | Influenza A virus (A/Chicken/Yunnan/447/05(H5N1)) |
| DQ095744 | 2 (PB1) | Influenza A virus (A/Chicken/Yunnan/447/05(H5N1)) |
| DQ095704 | 8 (NS) | Influenza A virus (A/Chicken/Yunnan/447/05(H5N1)) |
| DQ095664 | 6 (NA) | Influenza A virus (A/Chicken/Yunnan/447/05(H5N1)) |
| DQ095724 | 3 (PA) | Influenza A virus (A/Chicken/Yunnan/447/05(H5N1)) |
| DQ095684 | 5 (NP) | Influenza A virus (A/Chicken/Yunnan/447/05(H5N1)) |
| DQ095644 | 7 (MP) | Influenza A virus (A/Chicken/Yunnan/447/05(H5N1)) |
| DQ095624 | 4 (HA) | Influenza A virus (A/Chicken/Yunnan/447/05(H5N1)) |
| DQ095625 | 4 (HA) | Influenza A virus (A/Chicken/Yunnan/493/05(H5N1)) |
| DQ095645 | 7 (MP) | Influenza A virus (A/Chicken/Yunnan/493/05(H5N1)) |
| DQ095685 | 5 (NP) | Influenza A virus (A/Chicken/Yunnan/493/05(H5N1)) |
| DQ095725 | 3 (PA) | Influenza A virus (A/Chicken/Yunnan/493/05(H5N1)) |
| DQ095665 | 6 (NA) | Influenza A virus (A/Chicken/Yunnan/493/05(H5N1)) |
| DQ095705 | 8 (NS) | Influenza A virus (A/Chicken/Yunnan/493/05(H5N1)) |
| DQ095745 | 2 (PB1) | Influenza A virus (A/Chicken/Yunnan/493/05(H5N1)) |
| DQ095765 | 1 (PB2) | Influenza A virus (A/Chicken/Yunnan/493/05(H5N1)) |
| DQ321205 | 3 (PA) | Influenza A virus (A/chicken/Fujian/1042/2005(H5N1)) |
| DQ321139 | 8 (NS) | Influenza A virus (A/chicken/Fujian/1042/2005(H5N1)) |
| DQ320810 | 1 (PB2) | Influenza A virus (A/chicken/Fujian/1042/2005(H5N1)) |
| DQ320876 | 4 (HA) | Influenza A virus (A/chicken/Fujian/1042/2005(H5N1)) |
| DQ320942 | 7 (MP) | Influenza A virus (A/chicken/Fujian/1042/2005(H5N1)) |
| DQ321008 | 6 (NA) | Influenza A virus (A/chicken/Fujian/1042/2005(H5N1)) |
| DQ321073 | 5 (NP) | Influenza A virus (A/chicken/Fujian/1042/2005(H5N1)) |
| DQ321271 | 2 (PB1) | Influenza A virus (A/chicken/Fujian/1042/2005(H5N1)) |
| DQ095616 | 4 (HA) | Influenza A virus (A/Brown-headed Gull/Qinghai/3/05(H5N1)) |
| DQ095716 | 3 (PA) | Influenza A virus (A/Brown-headed Gull/Qinghai/3/05(H5N1)) |
| DQ095676 | 5 (NP) | Influenza A virus (A/Brown-headed Gull/Qinghai/3/05(H5N1)) |
| DQ095636 | 7 (MP) | Influenza A virus (A/Brown-headed Gull/Qinghai/3/05(H5N1)) |
| DQ095756 | 1 (PB2) | Influenza A virus (A/Brown-headed Gull/Qinghai/3/05(H5N1)) |
| DQ095736 | 2 (PB1) | Influenza A virus (A/Brown-headed Gull/Qinghai/3/05(H5N1)) |
| DQ095696 | 8 (NS) | Influenza A virus (A/Brown-headed Gull/Qinghai/3/05(H5N1)) |
| DQ095656 | 6 (NA) | Influenza A virus (A/Brown-headed Gull/Qinghai/3/05(H5N1)) |
| CY014167 | 1 (PB2) | Influenza A virus (A/Indonesia/5/2005(H5N1)) |
| CY014170 | 2 (PB1) | Influenza A virus (A/Indonesia/5/2005(H5N1)) |
| CY014171 | 3 (PA) | Influenza A virus (A/Indonesia/5/2005(H5N1)) |
| CY014172 | 5 (NP) | Influenza A virus (A/Indonesia/5/2005(H5N1)) |
| CY014173 | 7 (MP) | Influenza A virus (A/Indonesia/5/2005(H5N1)) |
| CY014174 | 8 (NS) | Influenza A virus (A/Indonesia/5/2005(H5N1)) |
| EF541395 | 6 (NA) | Influenza A virus (A/Indonesia/5/2005(H5N1)) |
| EF541394 | 4 (HA) | Influenza A virus (A/Indonesia/5/2005(H5N1)) |
| EU146634 | 1 (PB2) | Influenza A virus (A/Indonesia/7/2005(H5N1)) |
| EU146635 | 2 (PB1) | Influenza A virus (A/Indonesia/7/2005(H5N1)) |
| EU146639 | 8 (NS) | Influenza A virus (A/Indonesia/7/2005(H5N1)) |
| EU146637 | 5 (NP) | Influenza A virus (A/Indonesia/7/2005(H5N1)) |
| EU146636 | 3 (PA) | Influenza A virus (A/Indonesia/7/2005(H5N1)) |
| EU146633 | 6 (NA) | Influenza A virus (A/Indonesia/7/2005(H5N1)) |
| EU146632 | 4 (HA) | Influenza A virus (A/Indonesia/7/2005(H5N1)) |
| EU146638 | 7 (MP) | Influenza A virus (A/Indonesia/7/2005(H5N1)) |
| CY014269 | 1 (PB2) | Influenza A virus (A/Indonesia/CDC594/2006(H5N1)) |
| CY014270 | 2 (PB1) | Influenza A virus (A/Indonesia/CDC594/2006(H5N1)) |
| CY014271 | 3 (PA) | Influenza A virus (A/Indonesia/CDC594/2006(H5N1)) |
| CY014272 | 4 (HA) | Influenza A virus (A/Indonesia/CDC594/2006(H5N1)) |
| CY014273 | 5 (NP) | Influenza A virus (A/Indonesia/CDC594/2006(H5N1)) |
| CY014274 | 6 (NA) | Influenza A virus (A/Indonesia/CDC594/2006(H5N1)) |
| CY014275 | 7 (MP) | Influenza A virus (A/Indonesia/CDC594/2006(H5N1)) |
| CY014276 | 8 (NS) | Influenza A virus (A/Indonesia/CDC594/2006(H5N1)) |
| CY014277 | 1 (PB2) | Influenza A virus (A/Indonesia/CDC595/2006(H5N1)) |
| CY014278 | 2 (PB1) | Influenza A virus (A/Indonesia/CDC595/2006(H5N1)) |
| CY014279 | 3 (PA) | Influenza A virus (A/Indonesia/CDC595/2006(H5N1)) |
| CY014280 | 4 (HA) | Influenza A virus (A/Indonesia/CDC595/2006(H5N1)) |
| CY014281 | 5 (NP) | Influenza A virus (A/Indonesia/CDC595/2006(H5N1)) |
| CY014282 | 6 (NA) | Influenza A virus (A/Indonesia/CDC595/2006(H5N1)) |
| CY014283 | 7 (MP) | Influenza A virus (A/Indonesia/CDC595/2006(H5N1)) |
| CY014284 | 8 (NS) | Influenza A virus (A/Indonesia/CDC595/2006(H5N1)) |
| GU052413 | 6 (NA) | Influenza A virus (A/chicken/Indonesia/11/2003(H5N1)) |
| GU052417 | 1 (PB2) | Influenza A virus (A/chicken/Indonesia/11/2003(H5N1)) |
| GU052416 | 2 (PB1) | Influenza A virus (A/chicken/Indonesia/11/2003(H5N1)) |
| GU052414 | 5 (NP) | Influenza A virus (A/chicken/Indonesia/11/2003(H5N1)) |
| GU052415 | 8 (NS) | Influenza A virus (A/chicken/Indonesia/11/2003(H5N1)) |
| GU052412 | 7 (MP) | Influenza A virus (A/chicken/Indonesia/11/2003(H5N1)) |
| GU052411 | 4 (HA) | Influenza A virus (A/chicken/Indonesia/11/2003(H5N1)) |
| GU186744 | 1 (PB2) | Influenza A virus (A/chicken/Indonesia/7/2003(H5N1)) |
| GU186743 | 2 (PB1) | Influenza A virus (A/chicken/Indonesia/7/2003(H5N1)) |
| GU186742 | 3 (PA) | Influenza A virus (A/chicken/Indonesia/7/2003(H5N1)) |
| GU186741 | 8 (NS) | Influenza A virus (A/chicken/Indonesia/7/2003(H5N1)) |
| GU186740 | 5 (NP) | Influenza A virus (A/chicken/Indonesia/7/2003(H5N1)) |
| EF473082 | 6 (NA) | Influenza A virus (A/chicken/Indonesia/7/2003(H5N1)) |
| EF473079 | 7 (MP) | Influenza A virus (A/chicken/Indonesia/7/2003(H5N1)) |
| EF473080 | 4 (HA) | Influenza A virus (A/chicken/Indonesia/7/2003(H5N1)) |
| FJ455874 | 4 (HA) | Influenza A virus (A/chicken/Sukhothai/NIAH114843/2008(H5N1)) |
| FJ750820 | 7 (MP) | Influenza A virus (A/chicken/Sukhothai/NIAH114843/2008(H5N1)) |
| FJ750821 | 6 (NA) | Influenza A virus (A/chicken/Sukhothai/NIAH114843/2008(H5N1)) |
| FJ750822 | 5 (NP) | Influenza A virus (A/chicken/Sukhothai/NIAH114843/2008(H5N1)) |
| FJ750823 | 8 (NS) | Influenza A virus (A/chicken/Sukhothai/NIAH114843/2008(H5N1)) |
| FJ750824 | 3 (PA) | Influenza A virus (A/chicken/Sukhothai/NIAH114843/2008(H5N1)) |
| FJ750825 | 2 (PB1) | Influenza A virus (A/chicken/Sukhothai/NIAH114843/2008(H5N1)) |
| FJ750826 | 1 (PB2) | Influenza A virus (A/chicken/Sukhothai/NIAH114843/2008(H5N1)) |
| CY098755 | 1 (PB2) | Influenza A virus (A/Hubei/1/2010(H5N1)) |
| CY098756 | 2 (PB1) | Influenza A virus (A/Hubei/1/2010(H5N1)) |
| CY098757 | 3 (PA) | Influenza A virus (A/Hubei/1/2010(H5N1)) |
| CY098758 | 4 (HA) | Influenza A virus (A/Hubei/1/2010(H5N1)) |
| CY098759 | 5 (NP) | Influenza A virus (A/Hubei/1/2010(H5N1)) |
| CY098760 | 6 (NA) | Influenza A virus (A/Hubei/1/2010(H5N1)) |
| CY098761 | 7 (MP) | Influenza A virus (A/Hubei/1/2010(H5N1)) |
| CY098762 | 8 (NS) | Influenza A virus (A/Hubei/1/2010(H5N1)) |
| HQ636461 | 4 (HA) | Influenza A virus (A/Hong Kong/6841/2010(H5N1)) |
| HQ636463 | 7 (MP) | Influenza A virus (A/Hong Kong/6841/2010(H5N1)) |
| HQ636462 | 6 (NA) | Influenza A virus (A/Hong Kong/6841/2010(H5N1)) |
| HQ652626 | 5 (NP) | Influenza A virus (A/Hong Kong/6841/2010(H5N1)) |
| HQ652627 | 8 (NS) | Influenza A virus (A/Hong Kong/6841/2010(H5N1)) |
| HQ652628 | 3 (PA) | Influenza A virus (A/Hong Kong/6841/2010(H5N1)) |
| HQ652629 | 2 (PB1) | Influenza A virus (A/Hong Kong/6841/2010(H5N1)) |
| HQ652630 | 1 (PB2) | Influenza A virus (A/Hong Kong/6841/2010(H5N1)) |
| AF036356 | 4 (HA) | Influenza A virus (A/HongKong/156/97(H5N1)) |
| AF036358 | 7 (MP) | Influenza A virus (A/HongKong/156/97(H5N1)) |
| AF036357 | 6 (NA) | Influenza A virus (A/HongKong/156/97(H5N1)) |
| AF036359 | 5 (NP) | Influenza A virus (A/HongKong/156/97(H5N1)) |
| AF036360 | 8 (NS) | Influenza A virus (A/HongKong/156/97(H5N1)) |
| AF036361 | 3 (PA) | Influenza A virus (A/HongKong/156/97(H5N1)) |
| AF036362 | 2 (PB1) | Influenza A virus (A/HongKong/156/97(H5N1)) |
| AF036363 | 1 (PB2) | Influenza A virus (A/HongKong/156/97(H5N1)) |
| HM172072 | 4 (HA) | Influenza A virus (A/Anhui/2/2005(H5N1)) |
| HM172120 | 7 (MP) | Influenza A virus (A/Anhui/2/2005(H5N1)) |
| HM172165 | 6 (NA) | Influenza A virus (A/Anhui/2/2005(H5N1)) |
| HM172213 | 5 (NP) | Influenza A virus (A/Anhui/2/2005(H5N1)) |
| HM172264 | 8 (NS) | Influenza A virus (A/Anhui/2/2005(H5N1)) |
| HM172310 | 3 (PA) | Influenza A virus (A/Anhui/2/2005(H5N1)) |
| HM172359 | 2 (PB1) | Influenza A virus (A/Anhui/2/2005(H5N1)) |
| HM172451 | 1 (PB2) | Influenza A virus (A/Anhui/2/2005(H5N1)) |
| FJ390061 | 4 (HA) | Influenza A virus (A/plateau pika/Qinghai/04/2007(H5N1)） |
| FJ390064 | 7 (MP) | Influenza A virus (A/plateau pika/Qinghai/04/2007(H5N1)） |
| FJ390063 | 6 (NA) | Influenza A virus (A/plateau pika/Qinghai/04/2007(H5N1)） |
| FJ390062 | 5 (NP) | Influenza A virus (A/plateau pika/Qinghai/04/2007(H5N1)） |
| FJ390065 | 8 (NS) | Influenza A virus (A/plateau pika/Qinghai/04/2007(H5N1)） |
| FJ390060 | 3 (PA) | Influenza A virus (A/plateau pika/Qinghai/04/2007(H5N1)） |
| FJ390059 | 2 (PB1) | Influenza A virus (A/plateau pika/Qinghai/04/2007(H5N1)） |
| FJ390058 | 1 (PB2) | Influenza A virus (A/plateau pika/Qinghai/04/2007(H5N1)） |
| EF456805 | 4 (HA) | Influenza A virus (A/Cambodia/JP52a/2005(H5N1)) |
| EF456793 | 6 (NA) | Influenza A virus (A/Cambodia/JP52a/2005(H5N1)) |
| FJ225472 | 4 (HA) | Influenza A virus (A/Cambodia/R0405050/2007(H5N1)) |
| HQ200572 | 7 (MP) | Influenza A virus (A/Cambodia/R0405050/2007(H5N1)) |
| FJ225473 | 6 (NA) | Influenza A virus (A/Cambodia/R0405050/2007(H5N1)) |
| HQ200568 | 5 (NP) | Influenza A virus (A/Cambodia/R0405050/2007(H5N1)) |
| HQ200573 | 8 (NS) | Influenza A virus (A/Cambodia/R0405050/2007(H5N1)) |
| HQ200571 | 3 (PA) | Influenza A virus (A/Cambodia/R0405050/2007(H5N1)) |
| HQ200570 | 2 (PB1) | Influenza A virus (A/Cambodia/R0405050/2007(H5N1)) |
| HQ200569 | 1 (PB2) | Influenza A virus (A/Cambodia/R0405050/2007(H5N1)) |
| AY623430 | 4 (HA) | Influenza A virus (A/chicken/Yichang/lung-1/04(H5N1)) |
| AY623431 | 6 (NA) | Influenza A virus (A/chicken/Yichang/lung-1/04(H5N1)) |
| EF587274 | 1 (PB2) | Influenza A virus (A/Beijing/01/2003(H5N1)) |
| EF587275 | 2 (PB1) | Influenza A virus (A/Beijing/01/2003(H5N1)) |
| EF587276 | 3 (PA) | Influenza A virus (A/Beijing/01/2003(H5N1)) |
| EF587277 | 4 (HA) | Influenza A virus (A/Beijing/01/2003(H5N1)) |
| EF587278 | 5 (NP) | Influenza A virus (A/Beijing/01/2003(H5N1)) |
| EF587279 | 6 (NA) | Influenza A virus (A/Beijing/01/2003(H5N1)) |
| EF587280 | 7 (MP) | Influenza A virus (A/Beijing/01/2003(H5N1)) |
| EF587281 | 8 (NS) | Influenza A virus (A/Beijing/01/2003(H5N1)) |
| HM172113 | 4 (HA) | Influenza A virus (A/chicken/Shanxi/10/2006(H5N1)) |
| HM172135 | 7 (MP) | Influenza A virus (A/chicken/Shanxi/10/2006(H5N1)) |
| HM172172 | 6 (NA) | Influenza A virus (A/chicken/Shanxi/10/2006(H5N1)) |
| HM172243 | 5 (NP) | Influenza A virus (A/chicken/Shanxi/10/2006(H5N1)) |
| HM172297 | 8 (NS) | Influenza A virus (A/chicken/Shanxi/10/2006(H5N1)) |
| HM172337 | 3 (PA) | Influenza A virus (A/chicken/Shanxi/10/2006(H5N1)) |
| HM172361 | 2 (PB1) | Influenza A virus (A/chicken/Shanxi/10/2006(H5N1)) |
| HM172440 | 1 (PB2) | Influenza A virus (A/chicken/Shanxi/10/2006(H5N1)) |
| DQ914811 | 2 (PB1) | Influenza A virus (A/chicken/Shanxi/2/2006(H5N1)) |
| DQ914812 | 1 (PB2) | Influenza A virus (A/chicken/Shanxi/2/2006(H5N1)) |
| DQ914813 | 3 (PA) | Influenza A virus (A/chicken/Shanxi/2/2006(H5N1)) |
| DQ914815 | 5 (NP) | Influenza A virus (A/chicken/Shanxi/2/2006(H5N1)) |
| DQ914816 | 6 (NA) | Influenza A virus (A/chicken/Shanxi/2/2006(H5N1)) |
| DQ914817 | 7 (MP) | Influenza A virus (A/chicken/Shanxi/2/2006(H5N1)) |
| DQ914818 | 8 (NS) | Influenza A virus (A/chicken/Shanxi/2/2006(H5N1)) |
| DQ914814 | 4 (HA) | Influenza A virus (A/chicken/Shanxi/2/2006(H5N1)) |
| HQ677023 | 4 (HA) | Influenza A virus (A/chicken/Huadong/4/2008(H5N1)) |
| HQ677024 | 6 (NA) | Influenza A virus (A/chicken/Huadong/4/2008(H5N1)) |
| JX523356 | 1 (PB2) | Influenza A virus (A/chicken/Huadong/4/2008(H5N1)) |
| JX523357 | 2 (PB1) | Influenza A virus (A/chicken/Huadong/4/2008(H5N1)) |
| JX523358 | 3 (PA) | Influenza A virus (A/chicken/Huadong/4/2008(H5N1)) |
| JX523359 | 5 (NP) | Influenza A virus (A/chicken/Huadong/4/2008(H5N1)) |
| JX523360 | 7 (MP) | Influenza A virus (A/chicken/Huadong/4/2008(H5N1)) |
| JX523361 | 8 (NS) | Influenza A virus (A/chicken/Huadong/4/2008(H5N1)) |
| HM172092 | 4 (HA) | Influenza A virus (A/chicken/Jiangsu/18/2008(H5N1)) |
| HM172134 | 7 (MP) | Influenza A virus (A/chicken/Jiangsu/18/2008(H5N1)) |
| HM172171 | 6 (NA) | Influenza A virus (A/chicken/Jiangsu/18/2008(H5N1)) |
| HM172234 | 5 (NP) | Influenza A virus (A/chicken/Jiangsu/18/2008(H5N1)) |
| HM172275 | 8 (NS) | Influenza A virus (A/chicken/Jiangsu/18/2008(H5N1)) |
| HM172352 | 3 (PA) | Influenza A virus (A/chicken/Jiangsu/18/2008(H5N1)) |
| HM172368 | 2 (PB1) | Influenza A virus (A/chicken/Jiangsu/18/2008(H5N1)) |
| HM172417 | 1 (PB2) | Influenza A virus (A/chicken/Jiangsu/18/2008(H5N1)) |
| KX160180 | 6 (NA) | Influenza A virus (A/chicken/Liaoning/S4092/2011(H5N1)) |
| KX160181 | 5 (NP) | Influenza A virus (A/chicken/Liaoning/S4092/2011(H5N1)) |
| KX160182 | 8 (NS) | Influenza A virus (A/chicken/Liaoning/S4092/2011(H5N1)) |
| KX160183 | 3 (PA) | Influenza A virus (A/chicken/Liaoning/S4092/2011(H5N1)) |
| KX160184 | 2 (PB1) | Influenza A virus (A/chicken/Liaoning/S4092/2011(H5N1)) |
| KX160185 | 1 (PB2) | Influenza A virus (A/chicken/Liaoning/S4092/2011(H5N1)) |
| KX160186 | 4 (HA) | Influenza A virus (A/chicken/Liaoning/S4092/2011(H5N1)) |
| KX160187 | 7 (MP) | Influenza A virus (A/chicken/Liaoning/S4092/2011(H5N1)) |
| JX534546 | 1 (PB2) | Influenza A virus (A/wild duck/Shandong/1/2011(H5N1)) |
| JX534547 | 2 (PB1) | Influenza A virus (A/wild duck/Shandong/1/2011(H5N1)) |
| JX534548 | 3 (PA) | Influenza A virus (A/wild duck/Shandong/1/2011(H5N1)) |
| JX534549 | 4 (HA) | Influenza A virus (A/wild duck/Shandong/1/2011(H5N1)) |
| JX534550 | 5 (NP) | Influenza A virus (A/wild duck/Shandong/1/2011(H5N1)) |
| JX534551 | 6 (NA) | Influenza A virus (A/wild duck/Shandong/1/2011(H5N1)) |
| JX534552 | 7 (MP) | Influenza A virus (A/wild duck/Shandong/1/2011(H5N1)) |
| JX534553 | 8 (NS) | Influenza A virus (A/wild duck/Shandong/1/2011(H5N1)) |
| AF144300 | 1 (PB2) | Influenza A virus (A/goose/Guangdong/1/1996(H5N1)) |
| AF144301 | 2 (PB1) | Influenza A virus (A/goose/Guangdong/1/1996(H5N1)) |
| AF144302 | 3 (PA) | Influenza A virus (A/goose/Guangdong/1/1996(H5N1)) |
| AF144303 | 5 (NP) | Influenza A virus (A/goose/Guangdong/1/1996(H5N1)) |
| AF144304 | 6 (NA) | Influenza A virus (A/goose/Guangdong/1/1996(H5N1)) |
| AF144305 | 4 (HA) | Influenza A virus (A/goose/Guangdong/1/1996(H5N1)) |
| AF144306 | 7 (MP) | Influenza A virus (A/goose/Guangdong/1/1996(H5N1)) |
| AF144307 | 8 (NS) | Influenza A virus (A/goose/Guangdong/1/1996(H5N1)) |
| DQ992636 | 1 (PB2) | Influenza A virus (A/duck/Fujian/11094/2005(H5N1)) |
| DQ992826 | 4 (HA) | Influenza A virus (A/duck/Fujian/11094/2005(H5N1)) |
| EF123962 | 2 (PB1) | Influenza A virus (A/duck/Fujian/11094/2005(H5N1)) |
| EF124113 | 7 (MP) | Influenza A virus (A/duck/Fujian/11094/2005(H5N1)) |
| EF124264 | 6 (NA) | Influenza A virus (A/duck/Fujian/11094/2005(H5N1)) |
| EF124415 | 5 (NP) | Influenza A virus (A/duck/Fujian/11094/2005(H5N1)) |
| EF124566 | 8 (NS) | Influenza A virus (A/duck/Fujian/11094/2005(H5N1)) |
| EF124717 | 3 (PA) | Influenza A virus (A/duck/Fujian/11094/2005(H5N1)) |
| DQ992565 | 1 (PB2) | Influenza A virus (A/munia/Hong Kong/2454/2006(H5N1)) |
| DQ992845 | 4 (HA) | Influenza A virus (A/munia/Hong Kong/2454/2006(H5N1)) |
| EF123891 | 2 (PB1) | Influenza A virus (A/munia/Hong Kong/2454/2006(H5N1)) |
| EF124042 | 7 (MP) | Influenza A virus (A/munia/Hong Kong/2454/2006(H5N1)) |
| EF124193 | 6 (NA) | Influenza A virus (A/munia/Hong Kong/2454/2006(H5N1)) |
| EF124344 | 5 (NP) | Influenza A virus (A/munia/Hong Kong/2454/2006(H5N1)) |
| EF124495 | 8 (NS) | Influenza A virus (A/munia/Hong Kong/2454/2006(H5N1)) |
| EF124646 | 3 (PA) | Influenza A virus (A/munia/Hong Kong/2454/2006(H5N1)) |
| DQ320845 | 1 (PB2) | Influenza A virus (A/duck/Hunan/1265/2005(H5N1)) |
| DQ320911 | 4 (HA) | Influenza A virus (A/duck/Hunan/1265/2005(H5N1)) |
| DQ320977 | 7 (MP) | Influenza A virus (A/duck/Hunan/1265/2005(H5N1)) |
| DQ321043 | 6 (NA) | Influenza A virus (A/duck/Hunan/1265/2005(H5N1)) |
| DQ321108 | 5 (NP) | Influenza A virus (A/duck/Hunan/1265/2005(H5N1)) |
| DQ321306 | 2 (PB1) | Influenza A virus (A/duck/Hunan/1265/2005(H5N1)) |
| DQ321240 | 3 (PA) | Influenza A virus (A/duck/Hunan/1265/2005(H5N1)) |
| DQ321174 | 8 (NS) | Influenza A virus (A/duck/Hunan/1265/2005(H5N1)) |
| AY651486 | 5 (NP) | Influenza A virus (A/Dk/Indonesia/MS/2004(H5N1)) |
| AY651375 | 7 (MP) | Influenza A virus (A/Dk/Indonesia/MS/2004(H5N1)) |
| AY651322 | 4 (HA) | Influenza A virus (A/Dk/Indonesia/MS/2004(H5N1)) |
| AY651434 | 6 (NA) | Influenza A virus (A/Dk/Indonesia/MS/2004(H5N1)) |
| AY651652 | 2 (PB1) | Influenza A virus (A/Dk/Indonesia/MS/2004(H5N1)) |
| AY651540 | 8 (NS) | Influenza A virus (A/Dk/Indonesia/MS/2004(H5N1)) |
| AY651706 | 1 (PB2) | Influenza A virus (A/Dk/Indonesia/MS/2004(H5N1)) |
| AY651600 | 3 (PA) | Influenza A virus (A/Dk/Indonesia/MS/2004(H5N1)) |
| EU146800 | 1 (PB2) | Influenza A virus (A/Indonesia/546bH/2006(H5N1)) |
| EU146799 | 2 (PB1) | Influenza A virus (A/Indonesia/546bH/2006(H5N1)) |
| EU146797 | 8 (NS) | Influenza A virus (A/Indonesia/546bH/2006(H5N1)) |
| EU146796 | 5 (NP) | Influenza A virus (A/Indonesia/546bH/2006(H5N1)) |
| EU146798 | 3 (PA) | Influenza A virus (A/Indonesia/546bH/2006(H5N1)) |
| EU146795 | 6 (NA) | Influenza A virus (A/Indonesia/546bH/2006(H5N1)) |
| EU146793 | 4 (HA) | Influenza A virus (A/Indonesia/546bH/2006(H5N1)) |
| EU146794 | 7 (MP) | Influenza A virus (A/Indonesia/546bH/2006(H5N1)) |
| CY017685 | 1 (PB2) | Influenza A virus (A/Indonesia/CDC887/2006(H5N1)) |
| CY017686 | 2 (PB1) | Influenza A virus (A/Indonesia/CDC887/2006(H5N1)) |
| CY017687 | 3 (PA) | Influenza A virus (A/Indonesia/CDC887/2006(H5N1)) |
| CY017688 | 4 (HA) | Influenza A virus (A/Indonesia/CDC887/2006(H5N1)) |
| CY017689 | 5 (NP) | Influenza A virus (A/Indonesia/CDC887/2006(H5N1)) |
| CY017690 | 6 (NA) | Influenza A virus (A/Indonesia/CDC887/2006(H5N1)) |
| CY017691 | 7 (MP) | Influenza A virus (A/Indonesia/CDC887/2006(H5N1)) |
| CY017692 | 8 (NS) | Influenza A virus (A/Indonesia/CDC887/2006(H5N1)) |
| CY017651 | 1 (PB2) | Influenza A virus (A/Indonesia/CDC940/2006(H5N1)) |
| CY017652 | 2 (PB1) | Influenza A virus (A/Indonesia/CDC940/2006(H5N1)) |
| CY017653 | 3 (PA) | Influenza A virus (A/Indonesia/CDC940/2006(H5N1)) |
| CY017654 | 4 (HA) | Influenza A virus (A/Indonesia/CDC940/2006(H5N1)) |
| CY017655 | 5 (NP) | Influenza A virus (A/Indonesia/CDC940/2006(H5N1)) |
| CY017656 | 6 (NA) | Influenza A virus (A/Indonesia/CDC940/2006(H5N1)) |
| CY017657 | 7 (MP) | Influenza A virus (A/Indonesia/CDC940/2006(H5N1)) |
| CY017658 | 8 (NS) | Influenza A virus (A/Indonesia/CDC940/2006(H5N1)) |
| AY651602 | 3 (PA) | Influenza A virus (A/chicken/Indonesia/PA/2003(H5N1)) |
| AY651705 | 1 (PB2) | Influenza A virus (A/chicken/Indonesia/PA/2003(H5N1)) |
| AY651653 | 2 (PB1) | Influenza A virus (A/chicken/Indonesia/PA/2003(H5N1)) |
| AY651541 | 8 (NS) | Influenza A virus (A/chicken/Indonesia/PA/2003(H5N1)) |
| AY651487 | 5 (NP) | Influenza A virus (A/chicken/Indonesia/PA/2003(H5N1)) |
| AY651376 | 7 (MP) | Influenza A virus (A/chicken/Indonesia/PA/2003(H5N1)) |
| AY651433 | 6 (NA) | Influenza A virus (A/chicken/Indonesia/PA/2003(H5N1)) |
| AY651320 | 4 (HA) | Influenza A virus (A/chicken/Indonesia/PA/2003(H5N1)) |
| AY737285 | 8 (NS) | Influenza A virus (A/chicken/Guangdong/191/04(H5N1)) |
| AY737286 | 1 (PB2) | Influenza A virus (A/chicken/Guangdong/191/04(H5N1)) |
| AY737287 | 2 (PB1) | Influenza A virus (A/chicken/Guangdong/191/04(H5N1)) |
| AY737288 | 3 (PA) | Influenza A virus (A/chicken/Guangdong/191/04(H5N1)) |
| AY737289 | 4 (HA) | Influenza A virus (A/chicken/Guangdong/191/04(H5N1)) |
| AY737290 | 5 (NP) | Influenza A virus (A/chicken/Guangdong/191/04(H5N1)) |
| AY737291 | 6 (NA) | Influenza A virus (A/chicken/Guangdong/191/04(H5N1)) |
| AY737292 | 7 (MP) | Influenza A virus (A/chicken/Guangdong/191/04(H5N1)) |
| DQ320844 | 1 (PB2) | Influenza A virus (A/chicken/Hunan/999/2005(H5N1)) |
| DQ320910 | 4 (HA) | Influenza A virus (A/chicken/Hunan/999/2005(H5N1)) |
| DQ320976 | 7 (MP) | Influenza A virus (A/chicken/Hunan/999/2005(H5N1)) |
| DQ321042 | 6 (NA) | Influenza A virus (A/chicken/Hunan/999/2005(H5N1)) |
| DQ321107 | 5 (NP) | Influenza A virus (A/chicken/Hunan/999/2005(H5N1)) |
| DQ321305 | 2 (PB1) | Influenza A virus (A/chicken/Hunan/999/2005(H5N1)) |
| DQ321239 | 3 (PA) | Influenza A virus (A/chicken/Hunan/999/2005(H5N1)) |
| DQ321173 | 8 (NS) | Influenza A virus (A/chicken/Hunan/999/2005(H5N1)) |
| DQ321138 | 8 (NS) | Influenza A virus (A/duck/Fujian/897/2005(H5N1)) |
| DQ321204 | 3 (PA) | Influenza A virus (A/duck/Fujian/897/2005(H5N1)) |
| DQ321270 | 2 (PB1) | Influenza A virus (A/duck/Fujian/897/2005(H5N1)) |
| DQ321072 | 5 (NP) | Influenza A virus (A/duck/Fujian/897/2005(H5N1)) |
| DQ321007 | 6 (NA) | Influenza A virus (A/duck/Fujian/897/2005(H5N1)) |
| DQ320941 | 7 (MP) | Influenza A virus (A/duck/Fujian/897/2005(H5N1)) |
| DQ320875 | 4 (HA) | Influenza A virus (A/duck/Fujian/897/2005(H5N1)) |
| DQ320809 | 1 (PB2) | Influenza A virus (A/duck/Fujian/897/2005(H5N1)) |
| AY609309 | 1 (PB2) | Influenza A virus (A/chicken/Guangdong/174/04(H5N1)) |
| AY609310 | 2 (PB1) | Influenza A virus (A/chicken/Guangdong/174/04(H5N1)) |
| AY609311 | 3 (PA) | Influenza A virus (A/chicken/Guangdong/174/04(H5N1)) |
| AY609312 | 4 (HA) | Influenza A virus (A/chicken/Guangdong/174/04(H5N1)) |
| AY609313 | 5 (NP) | Influenza A virus (A/chicken/Guangdong/174/04(H5N1)) |
| AY609314 | 6 (NA) | Influenza A virus (A/chicken/Guangdong/174/04(H5N1)) |
| AY609315 | 7 (MP) | Influenza A virus (A/chicken/Guangdong/174/04(H5N1)) |
| AY609316 | 8 (NS) | Influenza A virus (A/chicken/Guangdong/174/04(H5N1)) |
| AY676031 | 3 (PA) | Influenza A virus (A/chicken/Korea/es/2003(H5N1)) |
| AY676027 | 2 (PB1) | Influenza A virus (A/chicken/Korea/es/2003(H5N1)) |
| AY676035 | 4 (HA) | Influenza A virus (A/chicken/Korea/es/2003(H5N1)) |
| AY676023 | 1 (PB2) | Influenza A virus (A/chicken/Korea/es/2003(H5N1)) |
| AY676047 | 7 (MP) | Influenza A virus (A/chicken/Korea/es/2003(H5N1)) |
| AY676043 | 6 (NA) | Influenza A virus (A/chicken/Korea/es/2003(H5N1)) |
| AY676039 | 5 (NP) | Influenza A virus (A/chicken/Korea/es/2003(H5N1)) |
| AY676051 | 8 (NS) | Influenza A virus (A/chicken/Korea/es/2003(H5N1)) |
| AF509021 | 4 (HA) | Influenza A virus (A/Silky Chicken/Hong Kong/SF189/01 (H5N1)) |
| AF509045 | 7 (MP) | Influenza A virus (A/Silky Chicken/Hong Kong/SF189/01 (H5N1)) |
| AF509071 | 8 (NS) | Influenza A virus (A/Silky Chicken/Hong Kong/SF189/01 (H5N1)) |
| AF509097 | 6 (NA) | Influenza A virus (A/Silky Chicken/Hong Kong/SF189/01 (H5N1)) |
| AF509122 | 5 (NP) | Influenza A virus (A/Silky Chicken/Hong Kong/SF189/01 (H5N1)) |
| AF509148 | 1 (PB2) | Influenza A virus (A/Silky Chicken/Hong Kong/SF189/01 (H5N1)) |
| AF509174 | 2 (PB1) | Influenza A virus (A/Silky Chicken/Hong Kong/SF189/01 (H5N1)) |
| AF509200 | 3 (PA) | Influenza A virus (A/Silky Chicken/Hong Kong/SF189/01 (H5N1)) |
| AY651387 | 7 (MP) | Influenza A virus (A/Vietnam/1194/2004(H5N1)) |
| AY651498 | 5 (NP) | Influenza A virus (A/Vietnam/1194/2004(H5N1)) |
| AY651333 | 4 (HA) | Influenza A virus (A/Vietnam/1194/2004(H5N1)) |
| AY651445 | 6 (NA) | Influenza A virus (A/Vietnam/1194/2004(H5N1)) |
| AY651552 | 8 (NS) | Influenza A virus (A/Vietnam/1194/2004(H5N1)) |
| AY651664 | 2 (PB1) | Influenza A virus (A/Vietnam/1194/2004(H5N1)) |
| AY651718 | 1 (PB2) | Influenza A virus (A/Vietnam/1194/2004(H5N1)) |
| AY651610 | 3 (PA) | Influenza A virus (A/Vietnam/1194/2004(H5N1)) |
| DQ366332 | 6 (NA) | Influenza A virus (A/chicken/Guangxi/12/2004(H5N1)) |
| DQ366333 | 7 (MP) | Influenza A virus (A/chicken/Guangxi/12/2004(H5N1)) |
| DQ366334 | 8 (NS) | Influenza A virus (A/chicken/Guangxi/12/2004(H5N1)) |
| DQ366327 | 1 (PB2) | Influenza A virus (A/chicken/Guangxi/12/2004(H5N1)) |
| DQ366328 | 2 (PB1) | Influenza A virus (A/chicken/Guangxi/12/2004(H5N1)) |
| DQ366329 | 3 (PA) | Influenza A virus (A/chicken/Guangxi/12/2004(H5N1)) |
| DQ366330 | 4 (HA) | Influenza A virus (A/chicken/Guangxi/12/2004(H5N1)) |
| DQ366331 | 5 (NP) | Influenza A virus (A/chicken/Guangxi/12/2004(H5N1)) |
| AY818127 | 1 (PB2) | Influenza A virus (A/chicken/Vietnam/C58/04(H5N1)) |
| AY818130 | 2 (PB1) | Influenza A virus (A/chicken/Vietnam/C58/04(H5N1)) |
| AY818133 | 3 (PA) | Influenza A virus (A/chicken/Vietnam/C58/04(H5N1)) |
| AY818136 | 4 (HA) | Influenza A virus (A/chicken/Vietnam/C58/04(H5N1)) |
| AY818139 | 5 (NP) | Influenza A virus (A/chicken/Vietnam/C58/04(H5N1)) |
| AY818142 | 6 (NA) | Influenza A virus (A/chicken/Vietnam/C58/04(H5N1)) |
| AY818145 | 7 (MP) | Influenza A virus (A/chicken/Vietnam/C58/04(H5N1)) |
| AY818148 | 8 (NS) | Influenza A virus (A/chicken/Vietnam/C58/04(H5N1)) |
| AY585362 | 4 (HA) | Influenza A virus (A/duck/Guangdong/22/2002(H5N1)) |
| AY585490 | 2 (PB1) | Influenza A virus (A/duck/Guangdong/22/2002(H5N1)) |
| AY585469 | 3 (PA) | Influenza A virus (A/duck/Guangdong/22/2002(H5N1)) |
| AY585448 | 8 (NS) | Influenza A virus (A/duck/Guangdong/22/2002(H5N1)) |
| AY585427 | 5 (NP) | Influenza A virus (A/duck/Guangdong/22/2002(H5N1)) |
| AY585406 | 6 (NA) | Influenza A virus (A/duck/Guangdong/22/2002(H5N1)) |
| AY585385 | 7 (MP) | Influenza A virus (A/duck/Guangdong/22/2002(H5N1)) |
| AY585511 | 1 (PB2) | Influenza A virus (A/duck/Guangdong/22/2002(H5N1)) |
| DQ366341 | 7 (MP) | Influenza A virus (A/duck/Guangxi/13/2004(H5N1)) |
| DQ366340 | 6 (NA) | Influenza A virus (A/duck/Guangxi/13/2004(H5N1)) |
| DQ366335 | 1 (PB2) | Influenza A virus (A/duck/Guangxi/13/2004(H5N1)) |
| DQ366342 | 8 (NS) | Influenza A virus (A/duck/Guangxi/13/2004(H5N1)) |
| DQ366339 | 5 (NP) | Influenza A virus (A/duck/Guangxi/13/2004(H5N1)) |
| DQ366338 | 4 (HA) | Influenza A virus (A/duck/Guangxi/13/2004(H5N1)) |
| DQ366337 | 3 (PA) | Influenza A virus (A/duck/Guangxi/13/2004(H5N1)) |
| DQ366336 | 2 (PB1) | Influenza A virus (A/duck/Guangxi/13/2004(H5N1)) |
| AY585398 | 7 (MP) | Influenza A virus (A/duck/Guangxi/50/2001(H5N1)) |
| AY585516 | 1 (PB2) | Influenza A virus (A/duck/Guangxi/50/2001(H5N1)) |
| AY585411 | 6 (NA) | Influenza A virus (A/duck/Guangxi/50/2001(H5N1)) |
| AY585432 | 5 (NP) | Influenza A virus (A/duck/Guangxi/50/2001(H5N1)) |
| AY585453 | 8 (NS) | Influenza A virus (A/duck/Guangxi/50/2001(H5N1)) |
| AY585474 | 3 (PA) | Influenza A virus (A/duck/Guangxi/50/2001(H5N1)) |
| AY585375 | 4 (HA) | Influenza A virus (A/duck/Guangxi/50/2001(H5N1)) |
| AY585495 | 2 (PB1) | Influenza A virus (A/duck/Guangxi/50/2001(H5N1)) |
| DQ321154 | 8 (NS) | Influenza A virus (A/goose/Guangxi/2383/2004(H5N1)) |
| DQ321220 | 3 (PA) | Influenza A virus (A/goose/Guangxi/2383/2004(H5N1)) |
| DQ321286 | 2 (PB1) | Influenza A virus (A/goose/Guangxi/2383/2004(H5N1)) |
| DQ321088 | 5 (NP) | Influenza A virus (A/goose/Guangxi/2383/2004(H5N1)) |
| DQ321023 | 6 (NA) | Influenza A virus (A/goose/Guangxi/2383/2004(H5N1)) |
| DQ320957 | 7 (MP) | Influenza A virus (A/goose/Guangxi/2383/2004(H5N1)) |
| DQ320891 | 4 (HA) | Influenza A virus (A/goose/Guangxi/2383/2004(H5N1)) |
| DQ320825 | 1 (PB2) | Influenza A virus (A/goose/Guangxi/2383/2004(H5N1)) |
| DQ321216 | 3 (PA) | Influenza A virus (A/duck/Guangxi/1793/2004(H5N1)) |
| DQ321150 | 8 (NS) | Influenza A virus (A/duck/Guangxi/1793/2004(H5N1)) |
| DQ320887 | 4 (HA) | Influenza A virus (A/duck/Guangxi/1793/2004(H5N1)) |
| DQ320953 | 7 (MP) | Influenza A virus (A/duck/Guangxi/1793/2004(H5N1)) |
| DQ320821 | 1 (PB2) | Influenza A virus (A/duck/Guangxi/1793/2004(H5N1)) |
| DQ321084 | 5 (NP) | Influenza A virus (A/duck/Guangxi/1793/2004(H5N1)) |
| DQ321019 | 6 (NA) | Influenza A virus (A/duck/Guangxi/1793/2004(H5N1)) |
| DQ321282 | 2 (PB1) | Influenza A virus (A/duck/Guangxi/1793/2004(H5N1)) |
